# Supplementary material for: Synchronous RNA conformational changes trigger ordered phase transitions in crystals
Source: Nat Commun. 2021 Mar 19;12:1762. doi: 10.1038/s41467-021-21838-5 (PMC7979858; doi:10.1038/s41467-021-21838-5)
Supplement: Supplementary file 1 — Supplementary Information [file 41467_2021_21838_MOESM1_ESM.pdf]

Supplementary Figures

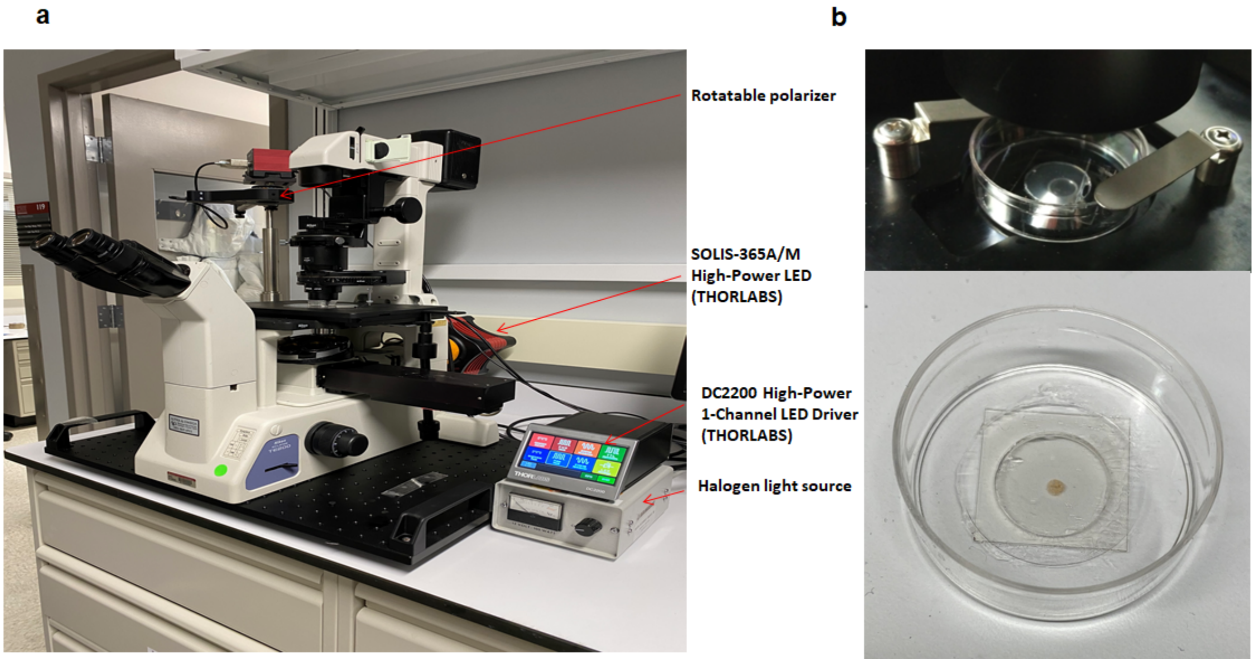

Supplementary Fig. 1. Video microscopy experiments.

a, A polarizing microscope was used to record time series for riboA crystals undergoing a phase transition in response to ligand mixing or UV illumination of crystals soaked with photo-caged (pc) ADE (Fig. **1a,** Supplementary Fig. **6b**). **b,** An optically transparent glass-bottom dish and the holder used for crystal imaging.

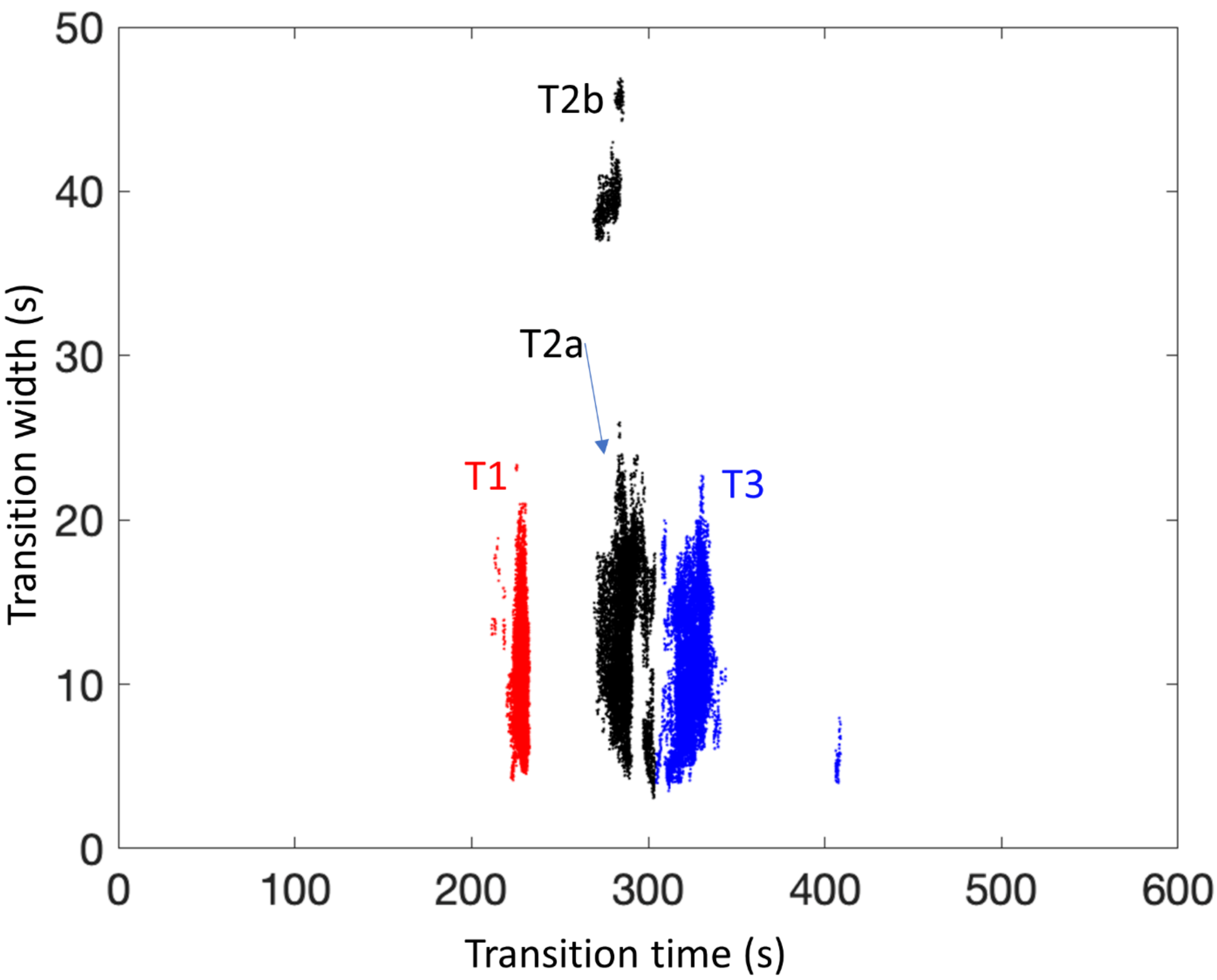

Supplementary Fig. 2. Transition halfwidth vs. transition time.

Distribution of peak half-width (*Y*-axis) and transition time (*X*-axis) for all (32,400) pixels in the ROI. Each peak in -$\partial I_i[(xy)_i t)\ ]/\partial t$ was fit to a Gaussian. K-means clustering was used to identify 3 clusters (red, black, and blue) corresponding to the three main transitions in the crystal, namely, transition 1 (T1), transition 2 (T2a&b) and transition 3 (T3) (Fig. **1c**). Each point represents a transition peak of each pixel. The 2D projection of the 3D plot is in Fig. **1c**.

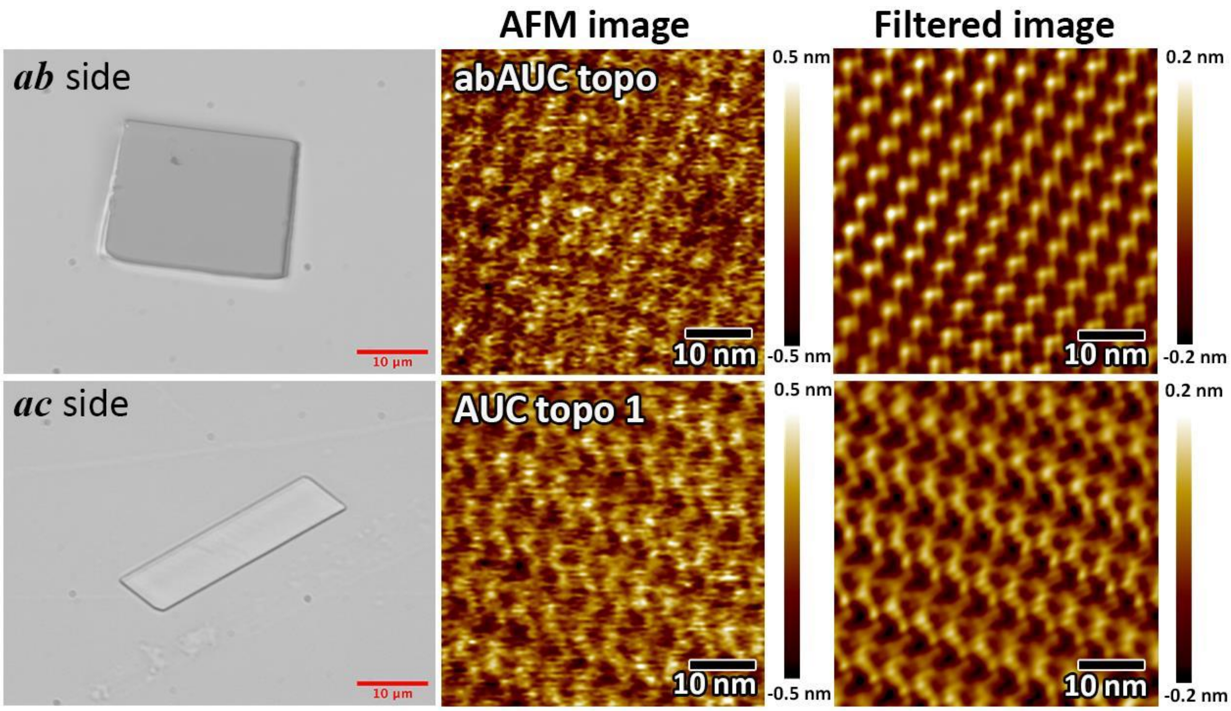

Supplementary Fig. 3. The optical microscope images of the *ab* face (square) and *ac* face (rectangular) riboA crystals and respective AFM topography and FFT filtered images.

The ***ac*** face crystals grow from 1:1000 concentration of crystal seeds, whereas 1:100 concentration of crystal seeds grow into ***ab*** face crystals on the spermidine treated mica surface (see **Methods**). AFM and FFT filtered images of ***ab*** apo unit cell topograph (abAUC topo) and *ac*AUC topo 1 have a size of 50 × 50 nm^2. The unit cell parameters for apo images of both ***ab*** and ***ac*** faces are listed in Supplementary Table **1**.

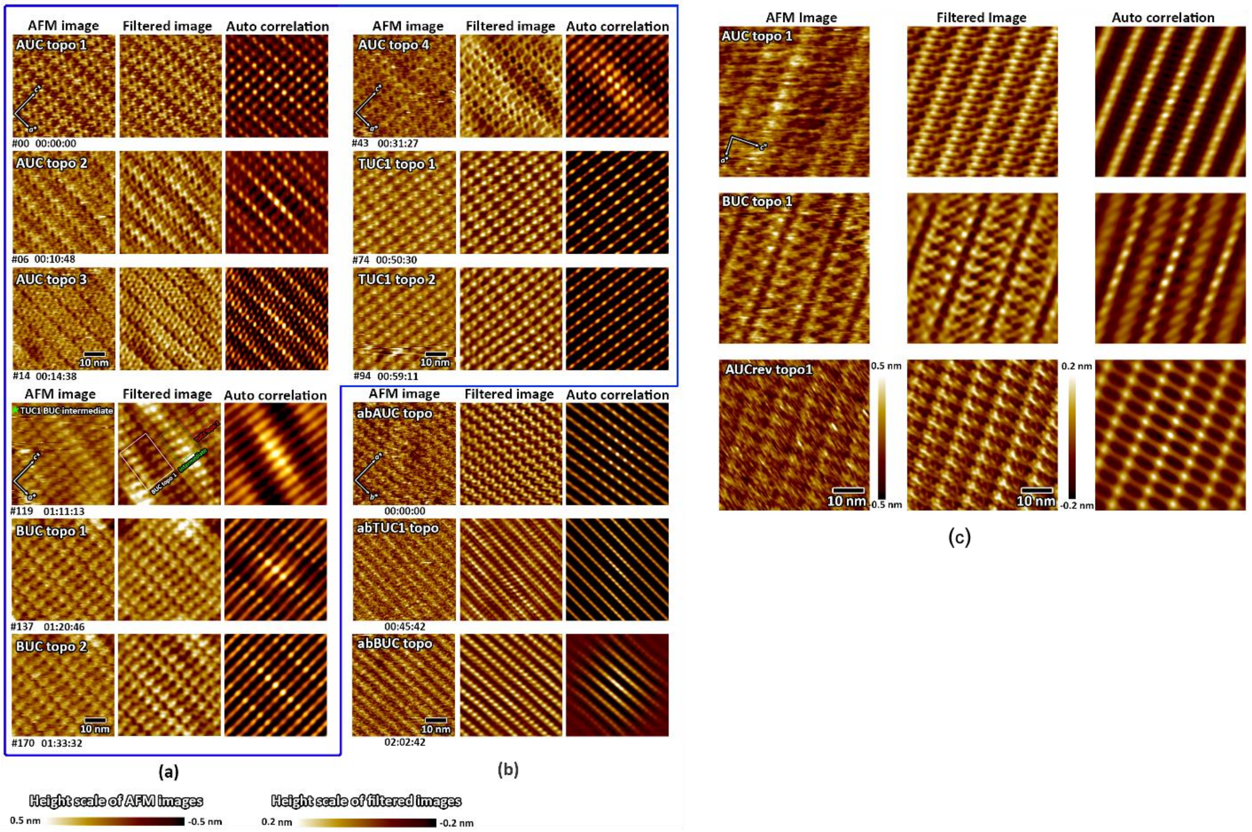

Supplementary Fig. 4. The raw and FFT filtered AFM images, and autocorrelation maps.
a, The panel of AFM topography images, corresponding FFT filtered images and auto correlation images of different phase transition stages in ***ac*** face crystals from apo to bound in the presence of 150µM adenine (Fig. **2a**). Apo unit cell topograph (AUC topo 1, AUC topo 2, AUC topo 3, AUC topo 4), trans unit cell topograph (TUC1 topo 1, TUC1 topo 2), TUC1 BUC intermediate, bound unit cell topograph (BUC topo 1, BUC topo 2).
b, The snapshots of AFM topography images, corresponding FFT filtered images and auto correlation images of abAUC topo, abTUC1 topo, and abBUC topo, recorded in ***ab*** face crystals in the presence of 50 µM adenine.
c, Forward and reverse phase transition in ***ac*** face crystal**.** FFT filtered images of phase transition from AUC topo 1 to BUC topo 1 in the presence of 50 µM ligand, and reversed phase transition to AUCrev topo 1 after extensive washing with ligand-free buffer. All AFM images are 50 × 50 nm^2 in size.

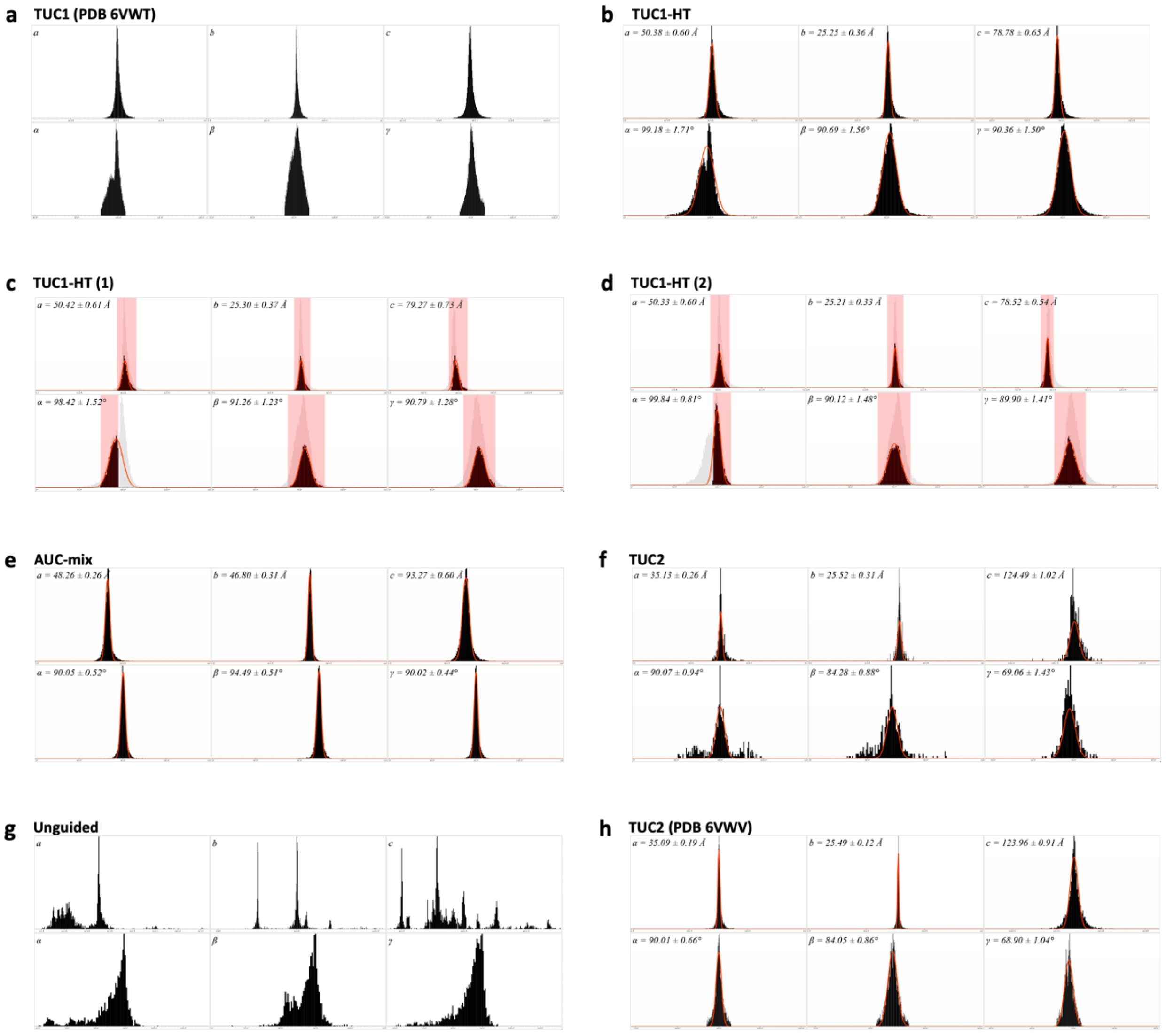

Supplementary Fig. 5. Histograms of indexing results for mixing delay times ranging from 25-175 s.

a,TUC1-indexed data using low tolerance (LT) indexing parameters. These data were used for crystal structure determination of PDB 6VWT. **b**, Data that could not be indexed as TUC1 using LT parameters were "reindexed" using XGANDALF with high tolerance (HT) parameters. **c-d**, TUC1 indexing yields a bifurcated peak for the α angle (98.4 and 99.8 °), where each sub-peak corresponds to a different fraction of the β angle peak. **e,** The remaining pool of data from **b**, which could not be indexed as TUC1, were then indexed in the same manner using AUC cell parameters. **f,** The remaining pool of data from **e**, which could not be indexed as AUC, were then indexed in the same manner using TUC2 cell parameters. **g,** The remaining pool of data from **f**, which could not be indexed as TUC2, were then indexed in the same manner using no guidance from unit cell parameters. **h,** TUC2-indexed data using LT parameters from a separate experiment in which riboA crystals were premixed with ligand and recorded from ~7–30 min post-mixing. These data were used for structure determination of PDB 6VWV.

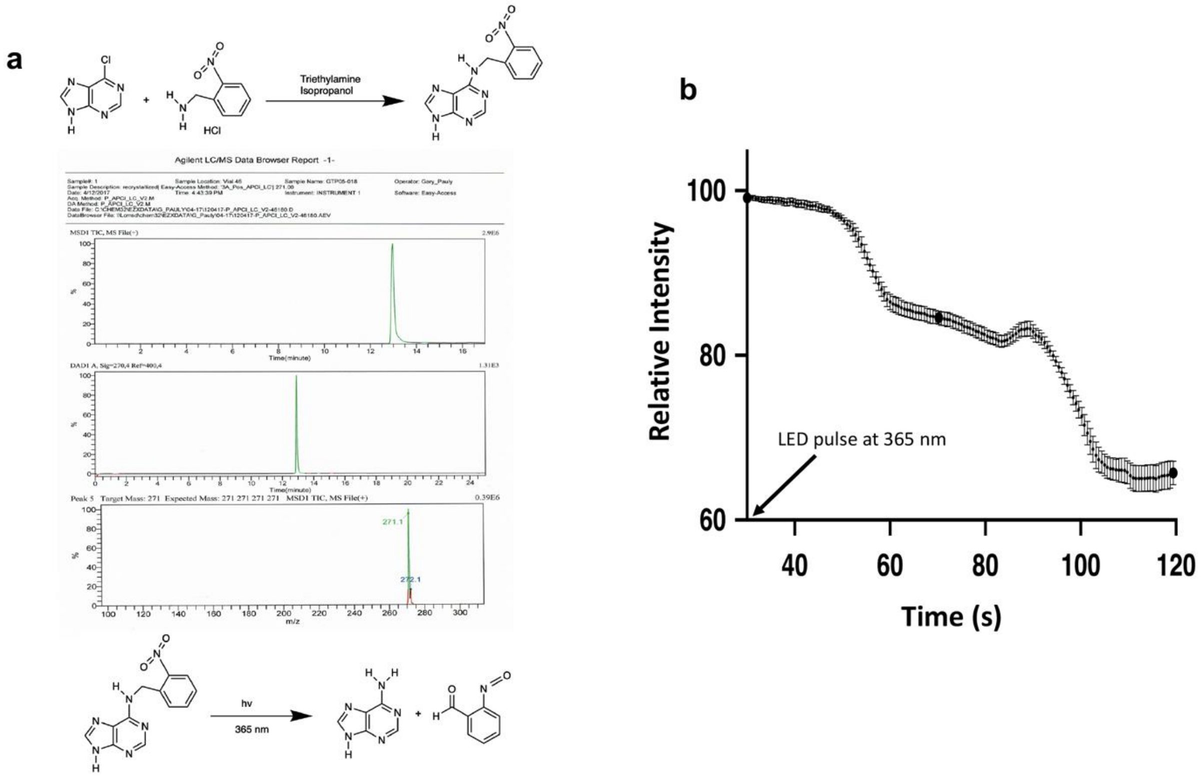

Supplementary Fig. 6. Photo-caged adenine and the phase transition curve.
a, Synthesis of photo-caged adenine (pcADE) (top), the LC-MS report for pcADE (middle), and the photolysis of pcADE at 365 nm (bottom). **b,** The time course of the birefringence changes plotted as the average of the relative birefringence intensity over five square areas of a riboA crystal, which was soaked with pcADE and illuminated with an LED pulse at 365 nm.

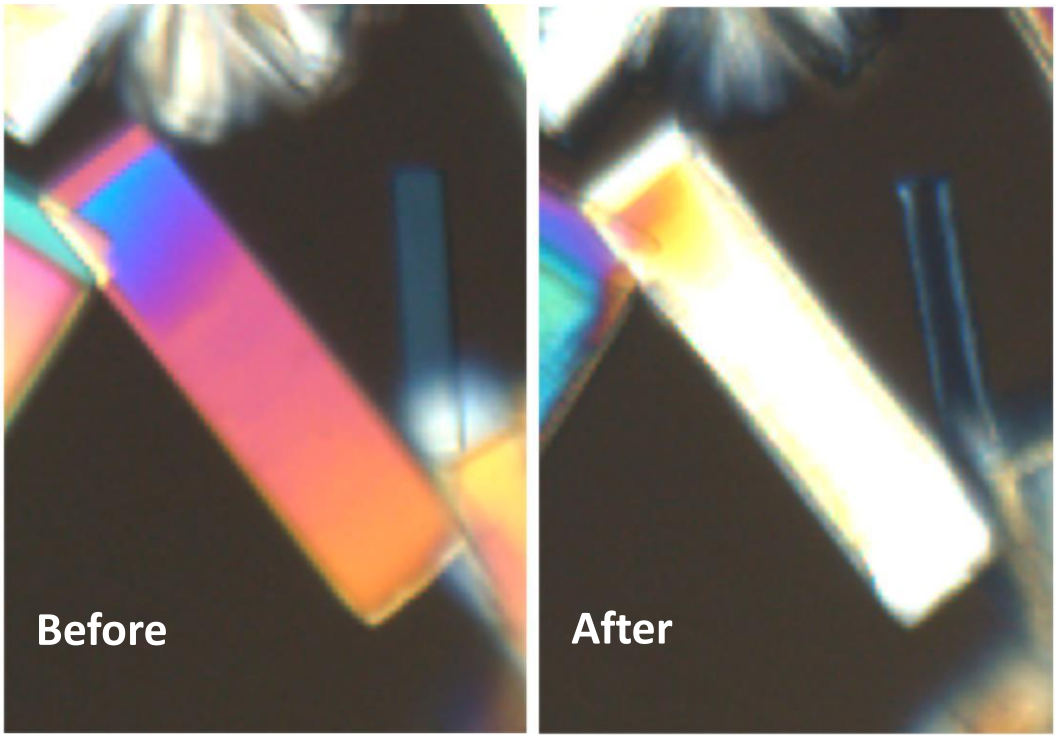

Supplementary Fig. 7. Polarized microscopy of tetracycline riboswitch crystals.

Polarized microscopy images showing the birefringence of a tetracycline riboswitch aptamer crystal before and after a lattice transition triggered by the addition of 10 mM ligand.

Supplementary Table 1. Unit cell parameters of crystal lattices derived from the phase transition experiments by AFM and XFEL

	Nomenclature	***a*** (Å)	***b*** (Å)	***c*** (Å)	***α*** (°)	***β*** (°)	***γ*** (°)
**AFM**							
Fig. 2a	AUC topo 1	49.6	-	94.2	-	95.7	-
	AUC topo 2	48.3	-	98.2	-	94.8	-
	AUC topo 3	48.6	-	93.8	-	96.9	-
	AUC topo 4	48	-	94.9	-	96.1	-
	TUC1 topo 1	49.3	-	78.2	-	91.1	-
	TUC1 topo 2	50.7	-	77.6	-	91.7	-
	TUC1 BUC intermediate [a]		-		-		-
	BUC topo 1	50.4	-	159.9	-	91.5	-
	BUC topo 2	49.9	-	155.9	-	89.3	-
Supplementary Fig. 4b	abAUC topo	49.6	46.2	-	-	-	91.5
	abTUC1 topo	50.3	24.1	-	-	-	92.7
	abBUC topo	50.9	24.8	-	-	-	90.2
Supplementary Fig. 4c	AUC topo 1	48.6	-	94.6	-	90.4	-
	BUC topo 1	48.4	-	157.4	-	91.5	-
	AUCrev topo 1	48.8	-	93.7	-	95.4	-
Supplementary Fig. 3	abAUC topo	48.4	44.4	-	-	-	88.2
	AUC topo 1	46.5	-	95.3	-	89.6	-
XFEL							
	AUC	48.3	46.9	93.4	90.0	94.5	90.0
	TUC1	50.3	25.2	78.7	99.3	90.6	90.2
	TUC2	35.1	25.5	124.0	90.0	84.1	68.9
	BUC	50.3	25.3	155.6	90.0	90.0	90.0

[a] The TUC1 BUC intermediate contains mixed lattices. Thus, unit cell parameters could not be derived.

Supplementary Table 2. Indexing of X-ray diffraction data.

	Delay Time (s)	Recorded Events	Hits	%	AUC-Indexed	%	TUC1-Indexed	%	Total	%
**Mixing**	0	398265	5901	1.5	8282	140.3	0	-	8282	140.3
	10	975304	11454	1.2	14321	124.8	0	-	14321	125.0
	25	1528534	14442	0.9	10357	71.3	1412	9.8	11769	81.5
	75	2538114	22522	0.9	1964	8.7	9634	42.8	11598	51.5
	100	1456206	18023	1.2	0	-	7900	43.8	7900	43.8
	125	572756	4486	0.8	0	-	2275	50.7	2275	50.7
	175	1202959	12967	1.1	0	-	6501	50.1	6501	50.1
	other [a]	910170	4465	0.5	2422	54.2	1128	25.3	3550	79.5
**TUC1** [b]	25-175	8208739	76905	0.9	-	-	28850	37.5	-	-

[a] The time delays for these data were uncertain due to experimental factors such as overlapping runs of data acquisition and unstable jetting of sample.
[b] These data were used for structure determination of PDB: 6VWT.

Supplementary Table 3. Reindexing with XGANDALF of diffraction patterns (25-175 s) with high-tolerance (HT) indexing parameters. [a]

	Delay Time (s)	Recorded Events	Hits	%	Indexed	%
**TUC1 (LT)**	25-175	8208739	76905	0.9	28850	37.5
**TUC1 (HT)**					16796	21.8
**AUC**					13436	17.5
**TUC2**					500	0.7
**Unguided**					1662	2.2
**AUC/TUC1** [b]					1558	2.0
**Not indexed**					18286	23.8
**Total Indexed**					62802	81.7

[a] XFEL mixing data (25-175s) that could not be indexed as TUC1 using low-tolerance parameters: peakfinding SNR=4 and cell tolerance of 7 % (axes) and 3° (angles), were reindexed using XGANDALF with high-tolerance settings: peakfinding SNR=3.5 and TUC1 cell parameters with a tolerance of 20 % (axes) and 10° (angles). This procedure was repeated iteratively for AUC, then TUC2, and finally no cell parameters, as described in Supplementary Fig. **5**.
[b] Hybrid patterns that could be indexed as either AUC or TUC1 using HT indexing parameters. Based on the 3D-merging (Fig. **3b**), the number of patterns containing diffraction from both AUC and TUC1 (but not necessarily indexable in both lattices) is predicted to be significantly higher.

Supplementary Table 4. Crystal data and structure refinement statistics.

Crystal Data	TUC1	TUC2
Space group	*P1*	*P1*
Unit cell parameters		
a, *b*, *c* (Å)	50.34, 25.20, 78.74	35.09, 25.49, 123.96
α, *β*, ɣ (°)	99.29, 90.55, 90.21	90.01, 84.05, 68.89
Matthews coefficient ($Å^3$/Da)	2.17	2.27
Solvent content (%)	43.4	45.7
Resolution (Å)	25.17–3.00 (3.11–3.00) [a]	27.53-3.00 (3.11–3.00)
No. recorded Events	8,208,739	250,579
No. diffraction patterns / Hit rate (%)	76,905 / 0.9	12,406 / 5.0
No. indexed patterns / Indexing Rate (%)	28,850 / 37.5	3770 / 30.4
No. unique reflections	7656 (803)	7980 (812)
Completeness	1.00 (1.00)	1.00 (1.00)
R_{split} (%)	7.34 (190.61)	23.43 (169.94)
SNR	13.49 (0.58)	3.46 (0.57)
Multiplicity	751.0 (207.7)	118.1 (74.6)
*CC**	0.9994 (0.9007)	0.9927 (0.7855)
**Structure Refinement**	**TUC1 (6VWT)**	**TUC2 (6VWV)**
Resolution range (Å)	25.17–3.04 (3.14–3.04)	27.00–3.00 (3.11–3.00)
Completeness for range	0.99 (0.96)	1.00 (0.99)
No. reflections used	7285 (747)	7952 (801)
No. reflections in test set	434 (40)	385 (44)
R_{work}	0.226 (0.390)	0.246 (0.392)
R_{free}	0.250 (0.327)	0.279 (0.438)
Wilson B ($Å^2$)	82.8	75.2
Average B ($Å^2$)	91.3	75.1
No. atoms	3034	3031
RNA	3006	3006
Ligand	20	20
Solvent	8	5
R.M.S. deviations		
Bond lengths (Å)	0.002	0.003
Bond angles (°)	0.520	0.940
Coordinate error estimate (Å)	0.33	0.38
All-atom clashscore	11.63	8.13

[a] Values in parentheses are for the highest resolution shell.

Supplementary Table 5. Crystal and data reduction statistics for data sets used in difference electron density map calculations.

Crystal Data	AUC-free	Combined AUC-10s	AUC-25s
Space group	*P21*	*P21*	*P21*
Unit cell parameters			
a, *b*, *c* (Å)	48.32, 46.86, 93.41	48.32, 46.86, 93.41	48.32, 46.86, 93.41
α, β, ɣ (°)	90.00, 94.51, 90.00	90.00, 94.51, 90.00	90.00, 94.51, 90.00
Matthews coefficient ($Å^3$/Da) / Solvent (%)	2.3 / 64.2	2.3 / 64.2	2.3 / 64.2
Resolution (Å)	24.09–2.30 (2.38–2.30) [a]	27.98–2.30 (2.38–2.30)	25.88-2.50 (2.59–2.50)
No. indexed patterns	29,052	38,107	10,357
No. unique reflections	18,806 (1,844)	18,812 (1,845)	14,692 (1,460)
Completeness	1.00 (1.00)	1.00 (1.00)	1.00 (1.00)
R_{split} (%)	11.04 (231.29)	8.75 (191.29)	16.09 (516.78)
SNR	6.53 (0.49)	7.79 (0.52)	4.76 (0.22)
Multiplicity	289.7 (50.2)	625.4 (265.5)	179.9 (76.9)
*CC**	0.998 (0.613)	0.999 (0.631)	0.996 (0.569)

[a] Values in parentheses are for the highest resolution shell.

Supplementary Table 6. Summary of results by various methods

	Pre-1st transition	**1st transition**	*Pre-2nd transition*	**2nd transition**	**3rd transition**
**PVM**		Sharp; >90% in 5 s		Relatively slow; Two different transition widths	Relatively slow; a broad transition width
**AFM**	Gradual continuous topological change (GCTC)	Lattice transition: AUC=>TUC1<br>*b*-axis: ~46Å to ~24Å<br>*c*-axis: ~95Å to ~78Å	GCTC	Intermediate between TUC1 and TUC2	TUC2 to BUC<br>*a*-axis: ~35Å to ~50Å<br>*c*-axis: ~124Å to ~156Å
**XFEL**	AUC lattice: apo1/apo2 to apo1/IB·Ade	Bragg diffraction of AUC, TUC1, AUC/TUC1 and unknown lattices<br>Lattice transition: AUC=>TUC1<br>*b*-axis: ~46Å to ~24Å<br>*c*-axis: ~95Å to ~78Å<br>apo1/apo2 convert to B·Ade-like		Bragg diffraction of TUC1 and TUC2<br>Lattice transition: TUC1=>TUC2<br>*a*-axis: ~50Å to ~35Å<br>*c*-axis: ~78Å to ~124Å	Bragg diffraction of BUC<br>Lattice transition: TUC1/TUC2=>BUC<br>*a*-axis: ~35Å to ~50Å<br>*c*-axis: ~124Å to ~156Å

Supplementary Movie 1. **Video of the riboA phase transitions triggered by ligand mixing.** Crystals suspended in 1.5 mL stabilization were added to the dish and the target crystal (***ac*** type) was centered and focused. The reaction was initiated by the addition of 1.5 mL ADE (2 mM). Video was recorded at 2456x1842 pixels. The time-lapsed of the video shown is ~24 m.

Supplementary Movie 2. The binary colored video of the riboA phase transitions triggered by ligand mixing. The $\partial I_i[(xy)_i t)\ ]/\partial t$ of each pixel vs. time is shown for the 4.5 x 4.5 μm^2 square sampling area with red indicating the centers of the transition in each pixel when $\partial I_i[(xy)_i t)\ ]/\partial t$ ~ 0, or blue elsewhere.

Supplementary Movie 3. Photoactivation of the riboswitch and physical manifestation of the phase transition in the riboA crystals. The manifestation of the phase transition, which is induced by uncaged adenine ligand with an LED light at 365 nm through conformational switching of the riboswitch upon ligand-binding.
