# Supplementary material for: Synchronous RNA conformational changes trigger ordered phase transitions in crystals
Source: Nat Commun. 2021 Mar 19;12:1762. doi: 10.1038/s41467-021-21838-5 (PMC7979858; doi:10.1038/s41467-021-21838-5)
Supplement: Supplementary file 3 — Description of Additional Supplementary Files [file 41467_2021_21838_MOESM3_ESM.pdf]

Description of Additional Supplementary Files

Supplementary Movie 1. **Video of the riboA phase transitions triggered by ligand mixing.** Crystals suspended in 1.5 mL stabilization were added to the dish and the target crystal (***ac*** type) was centered and focused. The reaction was initiated by the addition of 1.5 mL ADE (2 mM). Video was recorded at 2456x1842 pixels. The time-lapsed of the video shown is ~24 m.

Supplementary Movie 2. The binary colored video of the riboA phase transitions triggered by ligand mixing. The $\partial I_i[(xy)_i t)\ ]/\partial t$ of each pixel vs. time is shown for the 4.5 x 4.5 μm^2 square sampling area with red indicating the centers of the transition in each pixel when $\partial I_i[(xy)_i t)\ ]/\partial t$ ~ 0, or blue elsewhere.

Supplementary Movie 3. Photoactivation of the riboswitch and physical manifestation of the phase transition in the riboA crystals. The manifestation of the phase transition, which is induced by uncaged adenine ligand with an LED light at 365 nm through conformational switching of the riboswitch upon ligand-binding.
