# Supplementary figures and images for: Synchronous RNA conformational changes trigger ordered phase transitions in crystals
Source: Nat Commun. 2021 Mar 19;12:1762. doi: 10.1038/s41467-021-21838-5 (PMC7979858; doi:10.1038/s41467-021-21838-5)

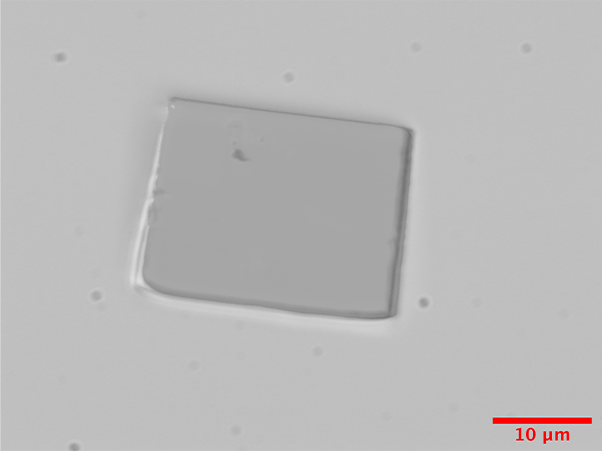

Supplement: Supplementary file 7 — Source Data [file 41467_2021_21838_MOESM7_ESM.zip › Source_Data/Supplementary_Figure3/ab side optical microscope image.tif]

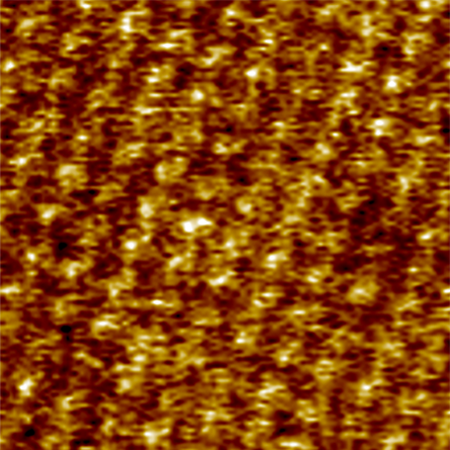

Supplement: Supplementary file 7 — Source Data [file 41467_2021_21838_MOESM7_ESM.zip › Source_Data/Supplementary_Figure3/abAUC topo AFM image.tif]

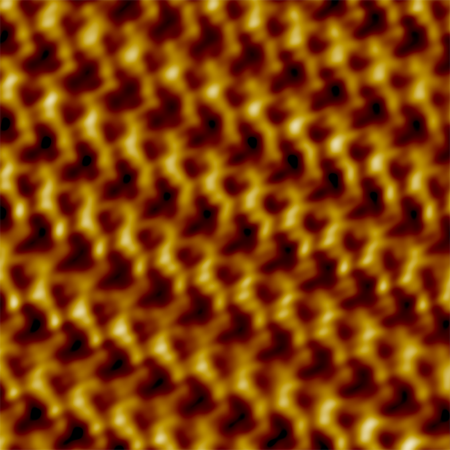

Supplement: Supplementary file 7 — Source Data [file 41467_2021_21838_MOESM7_ESM.zip › Source_Data/Supplementary_Figure3/AUC topo 1 filtered image.tif]

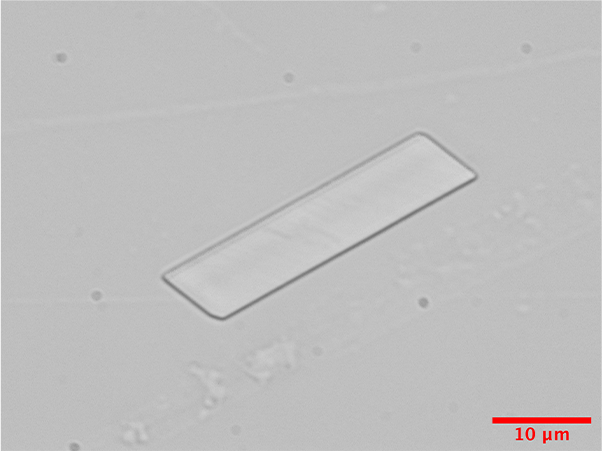

Supplement: Supplementary file 7 — Source Data [file 41467_2021_21838_MOESM7_ESM.zip › Source_Data/Supplementary_Figure3/ac side optical microscope image .tif]

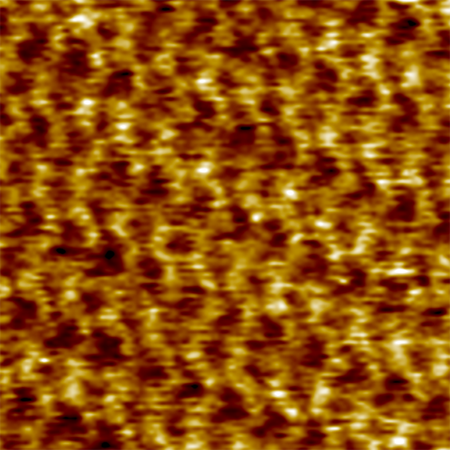

Supplement: Supplementary file 7 — Source Data [file 41467_2021_21838_MOESM7_ESM.zip › Source_Data/Supplementary_Figure3/AUC topo 1 AFM image.tif]

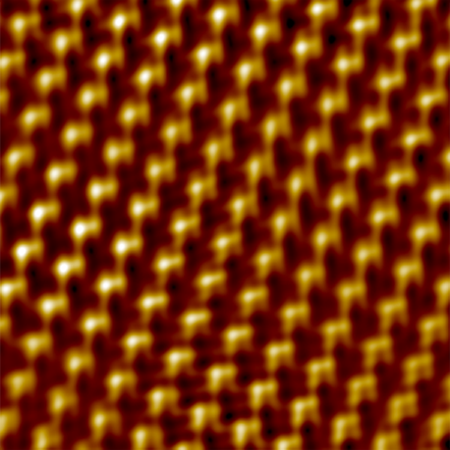

Supplement: Supplementary file 7 — Source Data [file 41467_2021_21838_MOESM7_ESM.zip › Source_Data/Supplementary_Figure3/abAUC topo filtered image.tif]

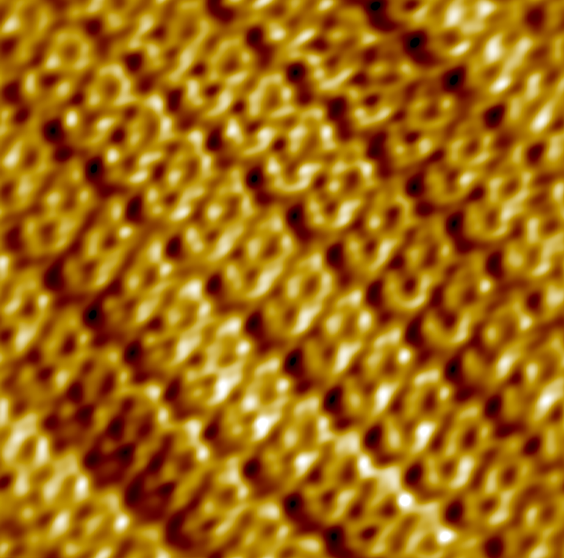

Supplement: Supplementary file 7 — Source Data [file 41467_2021_21838_MOESM7_ESM.zip › Source_Data/Figure2_Supplementary_Figure4a /Filtered images /AUC topo 3 Filtered image.tif]

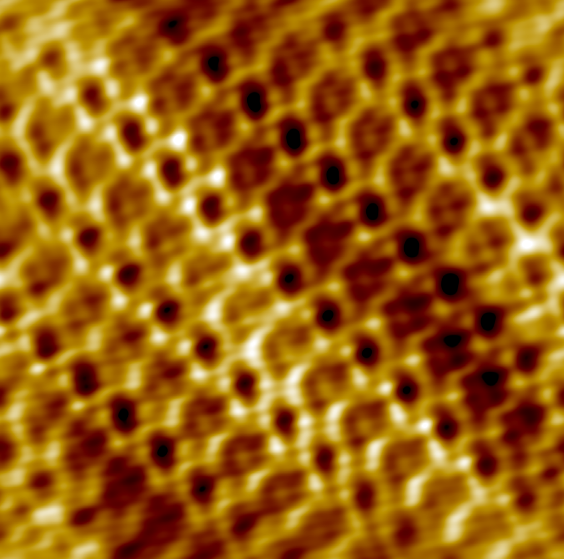

Supplement: Supplementary file 7 — Source Data [file 41467_2021_21838_MOESM7_ESM.zip › Source_Data/Figure2_Supplementary_Figure4a /Filtered images /AUC topo 4 Filtered image.tif]

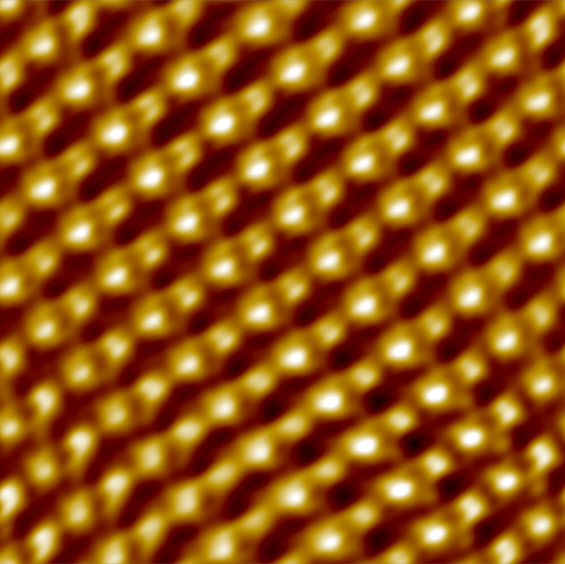

Supplement: Supplementary file 7 — Source Data [file 41467_2021_21838_MOESM7_ESM.zip › Source_Data/Figure2_Supplementary_Figure4a /Filtered images /TUC 1 topo 2 Filtered image.tif]

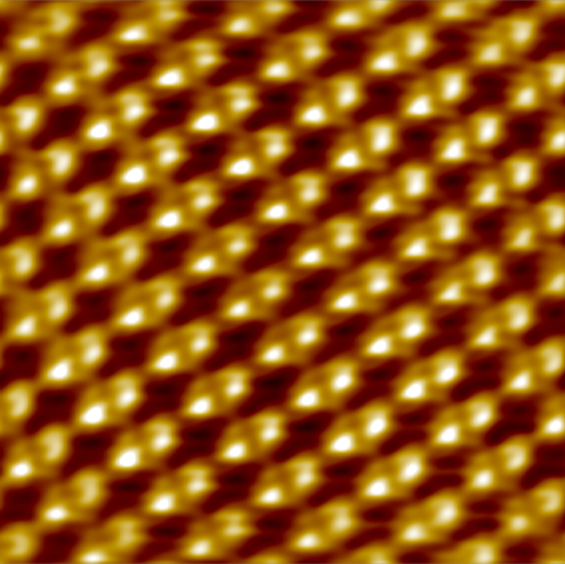

Supplement: Supplementary file 7 — Source Data [file 41467_2021_21838_MOESM7_ESM.zip › Source_Data/Figure2_Supplementary_Figure4a /Filtered images /TUC 1 topo 1 Filtered image.tif]

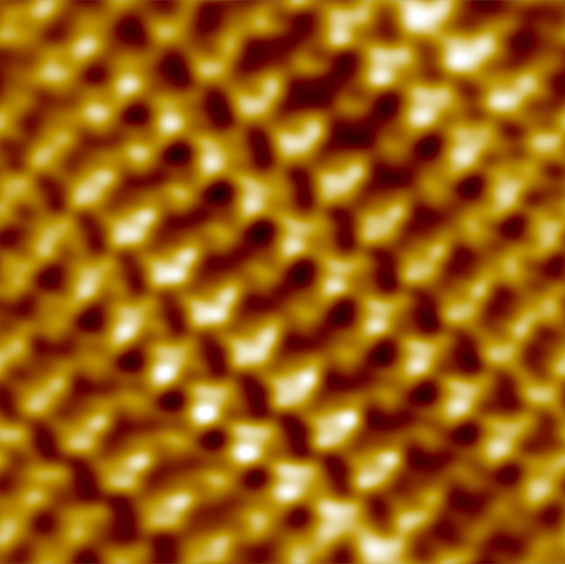

Supplement: Supplementary file 7 — Source Data [file 41467_2021_21838_MOESM7_ESM.zip › Source_Data/Figure2_Supplementary_Figure4a /Filtered images /BUC topo 2 Filtered image.tif]

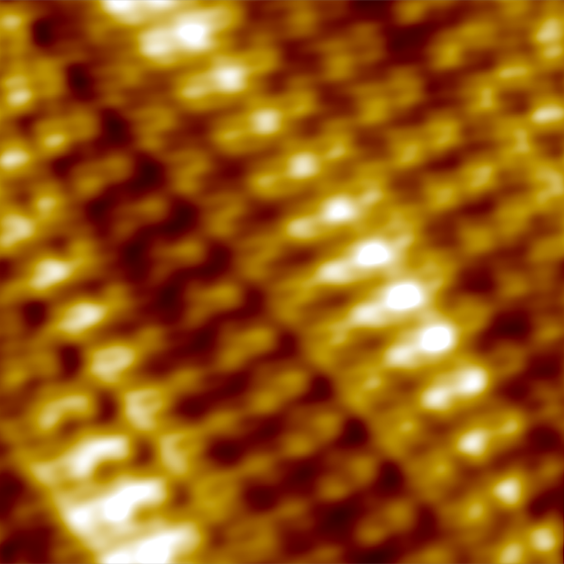

Supplement: Supplementary file 7 — Source Data [file 41467_2021_21838_MOESM7_ESM.zip › Source_Data/Figure2_Supplementary_Figure4a /Filtered images /TUC 1 BUC intermediate Filtered image.tif]

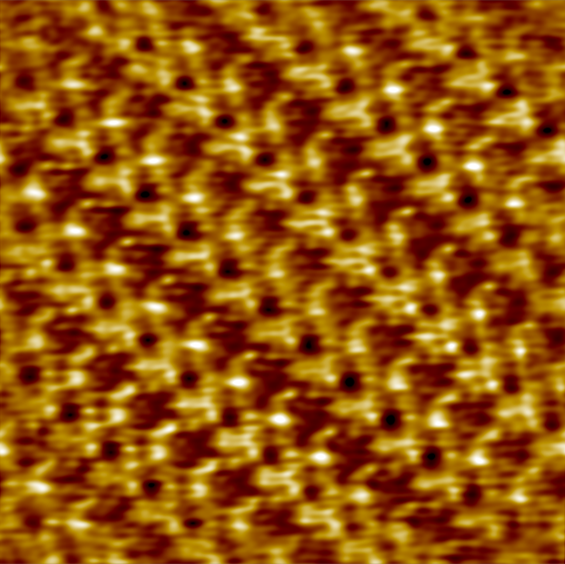

Supplement: Supplementary file 7 — Source Data [file 41467_2021_21838_MOESM7_ESM.zip › Source_Data/Figure2_Supplementary_Figure4a /Filtered images /AUC topo 1 Filtered image.tif]

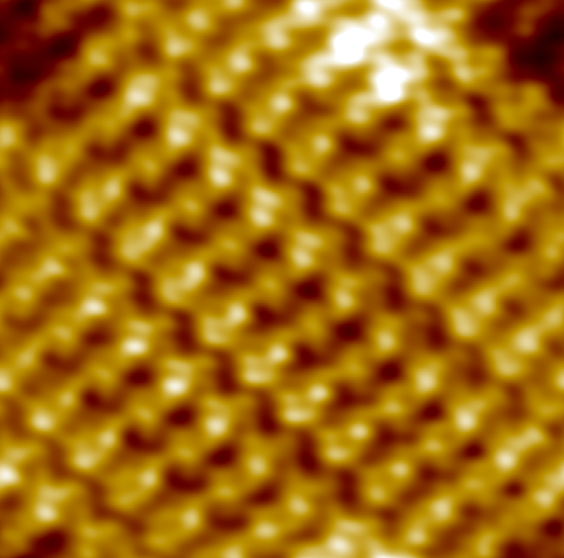

Supplement: Supplementary file 7 — Source Data [file 41467_2021_21838_MOESM7_ESM.zip › Source_Data/Figure2_Supplementary_Figure4a /Filtered images /BUC topo 1 Filtered image.tif]

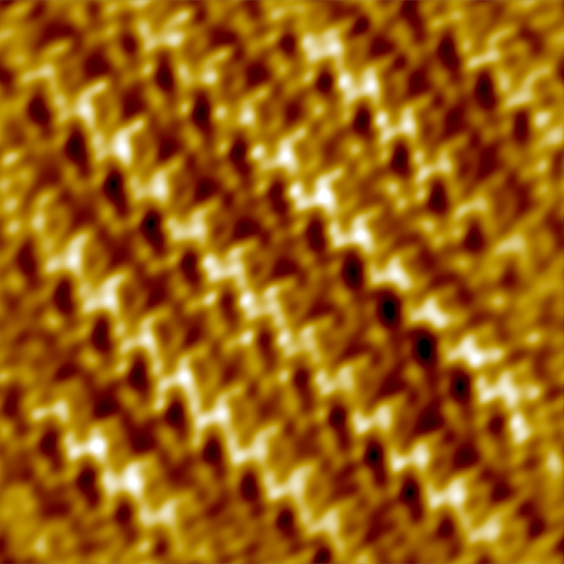

Supplement: Supplementary file 7 — Source Data [file 41467_2021_21838_MOESM7_ESM.zip › Source_Data/Figure2_Supplementary_Figure4a /Filtered images /AUC topo 2 Filtered image.tif]

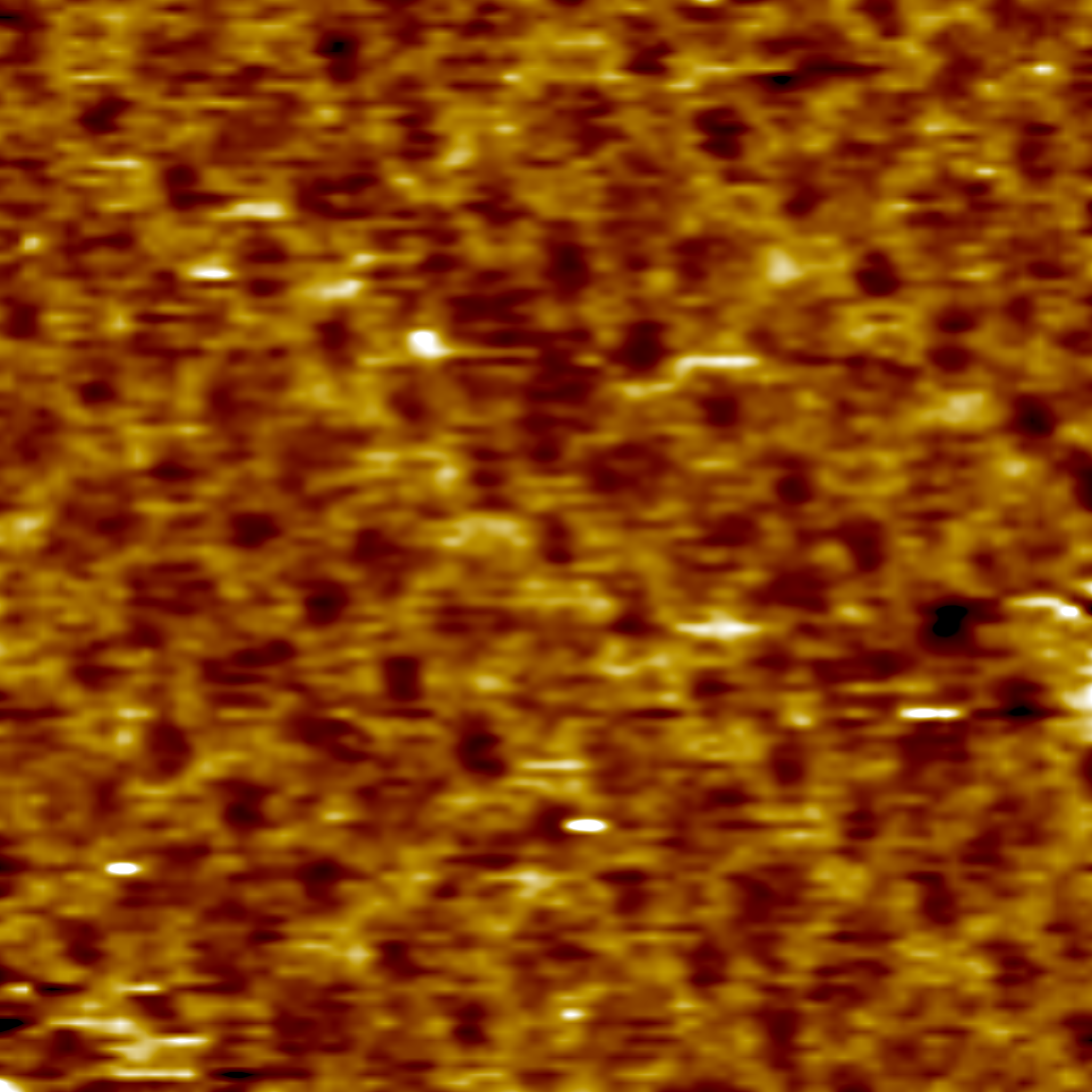

Supplement: Supplementary file 7 — Source Data [file 41467_2021_21838_MOESM7_ESM.zip › Source_Data/Figure2_Supplementary_Figure4a /Raw AFM images/AUC topo 4.tif]

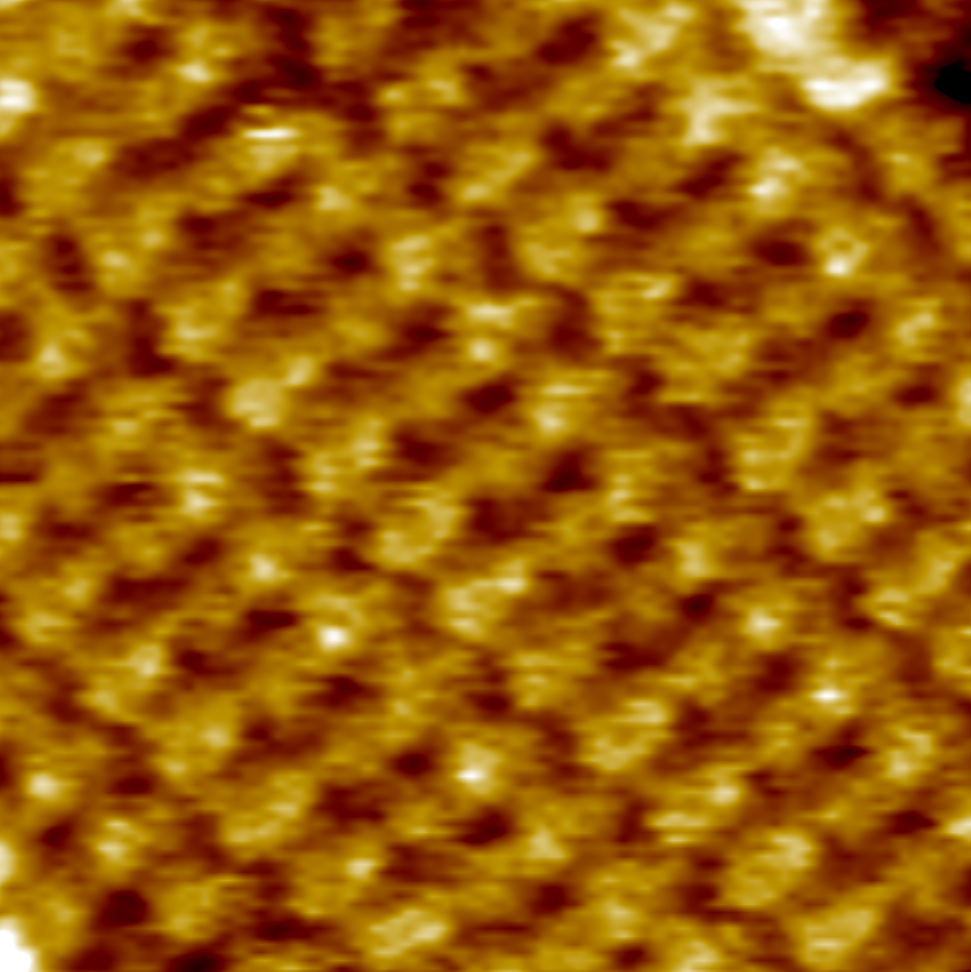

Supplement: Supplementary file 7 — Source Data [file 41467_2021_21838_MOESM7_ESM.zip › Source_Data/Figure2_Supplementary_Figure4a /Raw AFM images/BUC topo 1.tif]

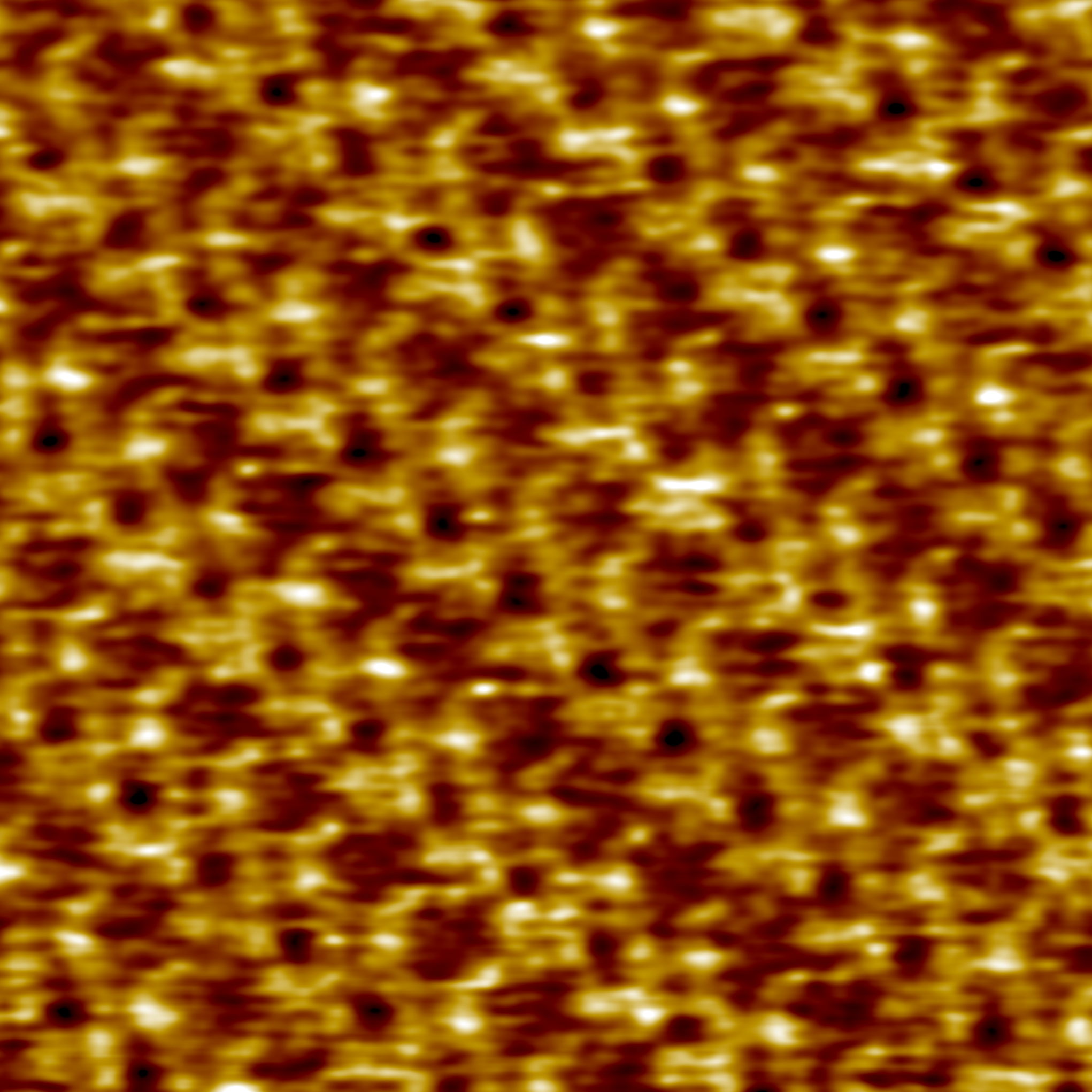

Supplement: Supplementary file 7 — Source Data [file 41467_2021_21838_MOESM7_ESM.zip › Source_Data/Figure2_Supplementary_Figure4a /Raw AFM images/AUC topo 1.tif]

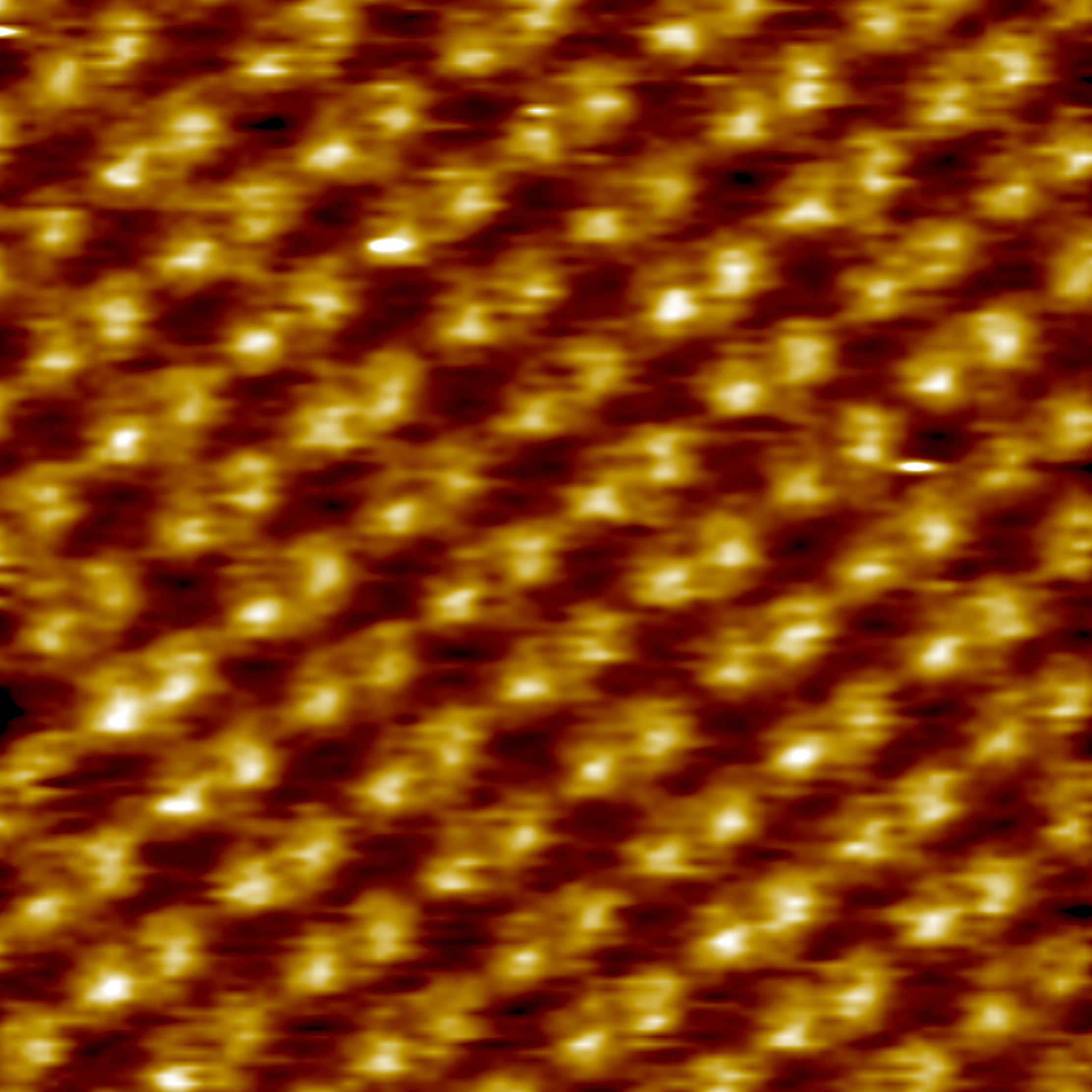

Supplement: Supplementary file 7 — Source Data [file 41467_2021_21838_MOESM7_ESM.zip › Source_Data/Figure2_Supplementary_Figure4a /Raw AFM images/TUC1 topo 1.tif]

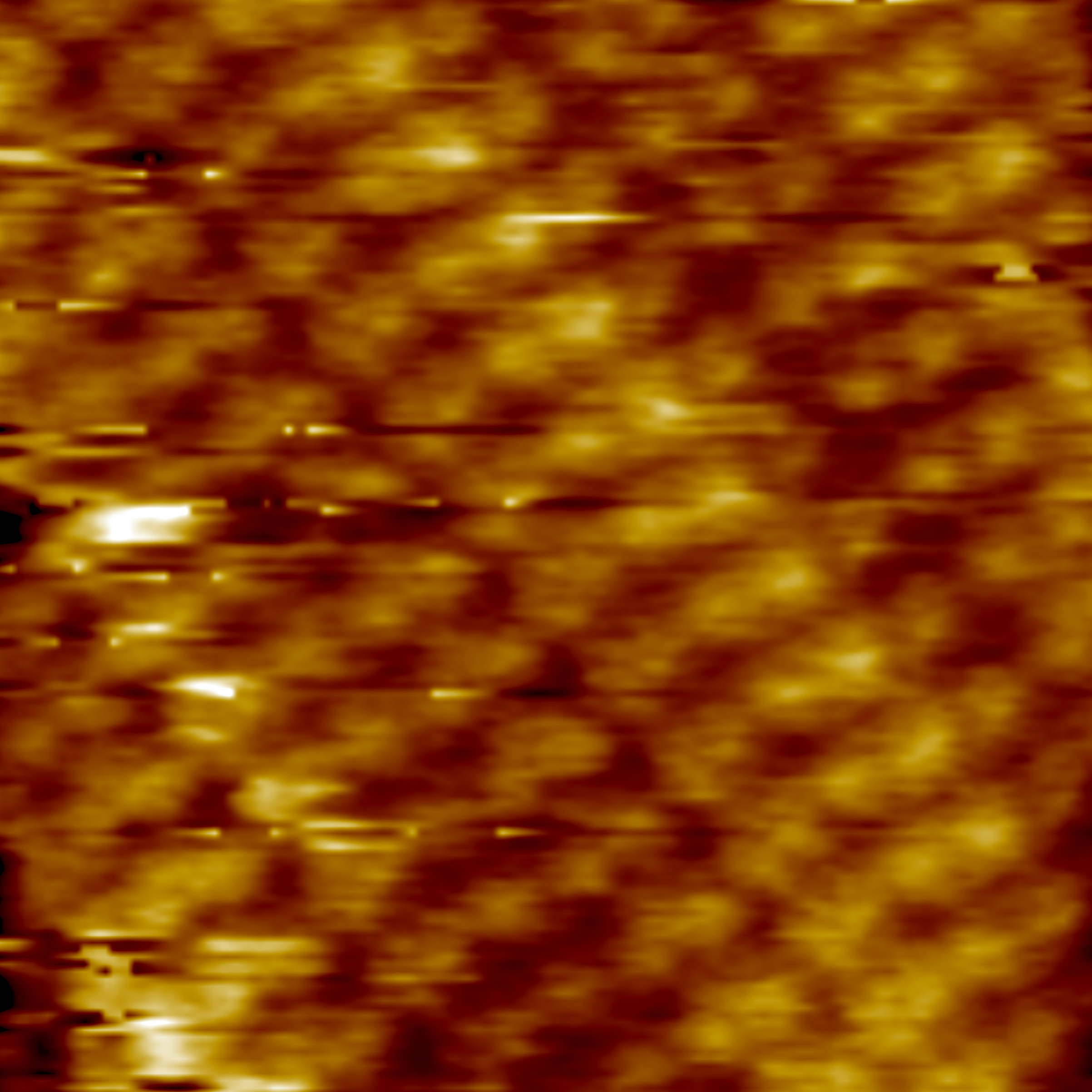

Supplement: Supplementary file 7 — Source Data [file 41467_2021_21838_MOESM7_ESM.zip › Source_Data/Figure2_Supplementary_Figure4a /Raw AFM images/TUC1 BUC intermediate.tif]

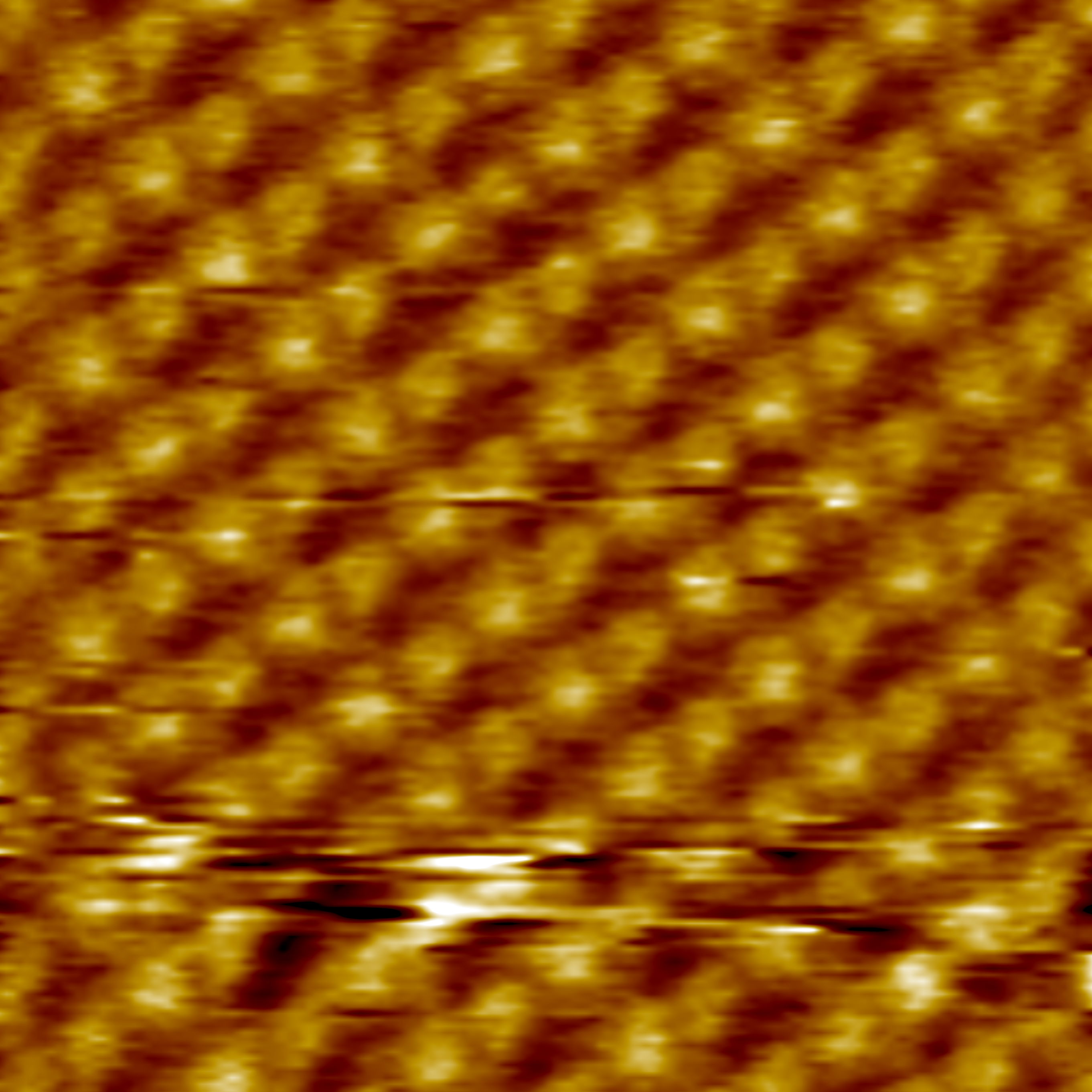

Supplement: Supplementary file 7 — Source Data [file 41467_2021_21838_MOESM7_ESM.zip › Source_Data/Figure2_Supplementary_Figure4a /Raw AFM images/TUC1 topo 2.tif]

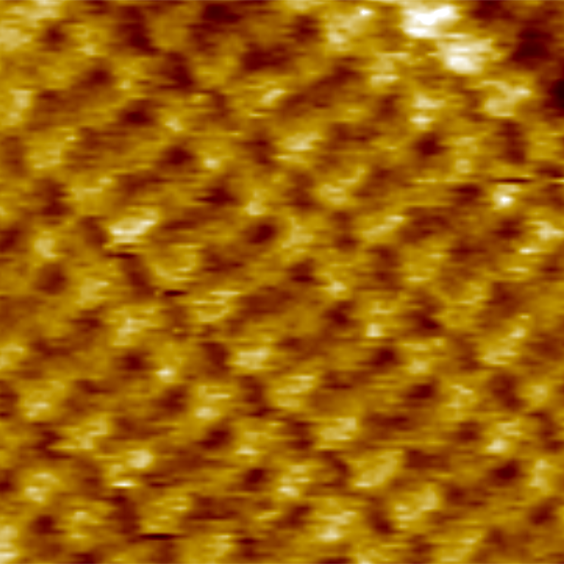

Supplement: Supplementary file 7 — Source Data [file 41467_2021_21838_MOESM7_ESM.zip › Source_Data/Figure2_Supplementary_Figure4a /Raw AFM images/BUC topo 2.tif]

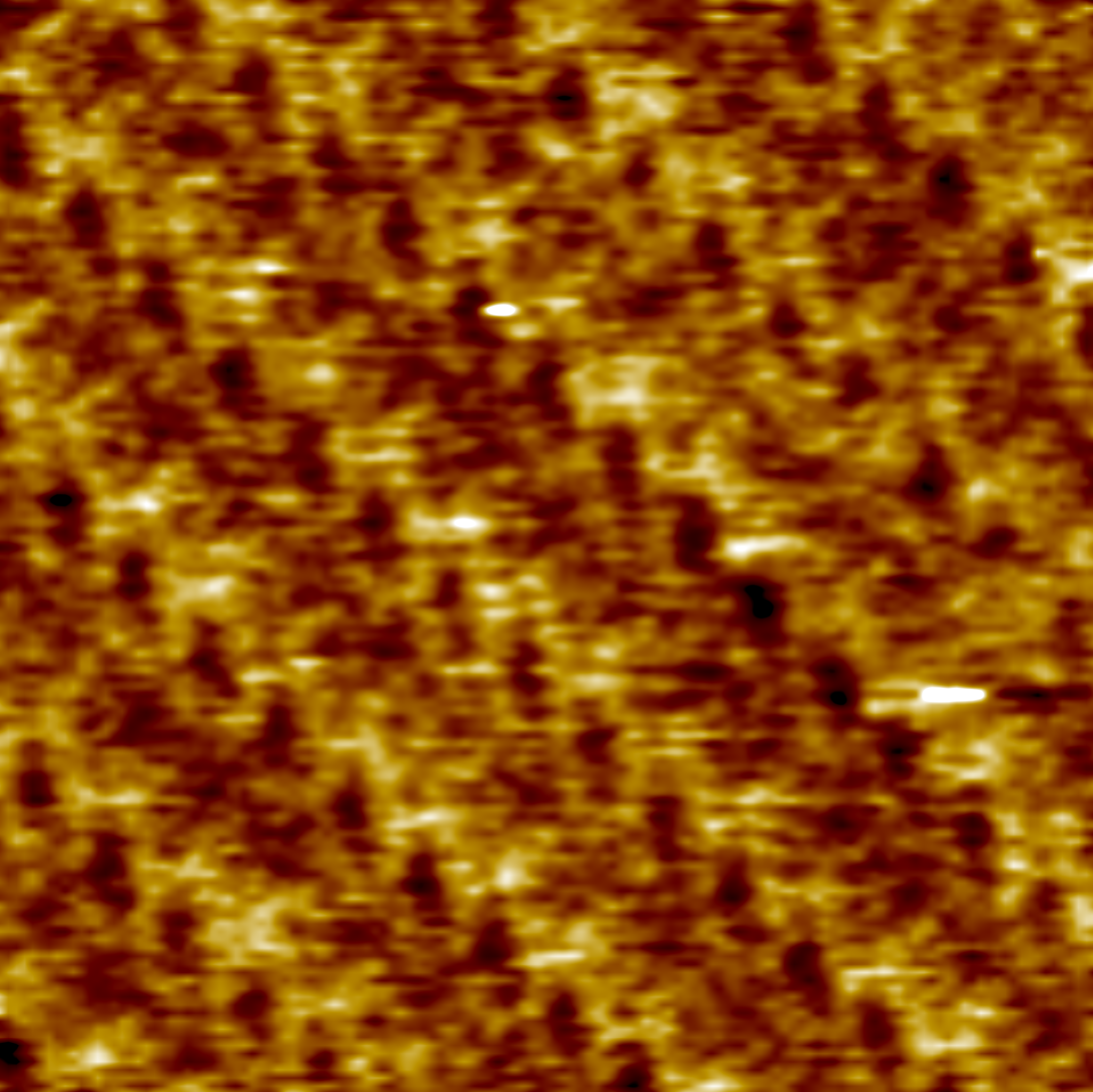

Supplement: Supplementary file 7 — Source Data [file 41467_2021_21838_MOESM7_ESM.zip › Source_Data/Figure2_Supplementary_Figure4a /Raw AFM images/AUC topo 2.tif]

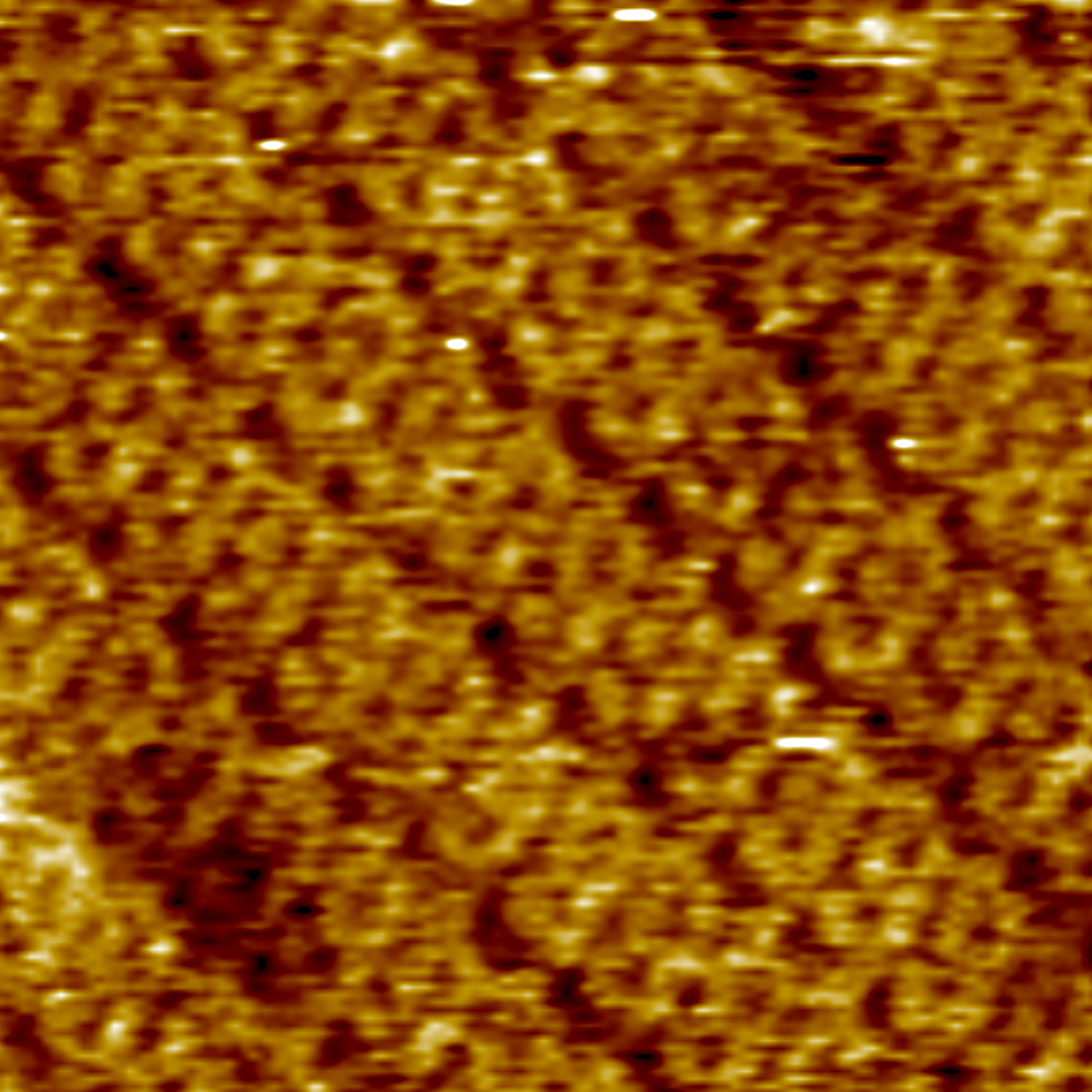

Supplement: Supplementary file 7 — Source Data [file 41467_2021_21838_MOESM7_ESM.zip › Source_Data/Figure2_Supplementary_Figure4a /Raw AFM images/AUC topo 3.tif]

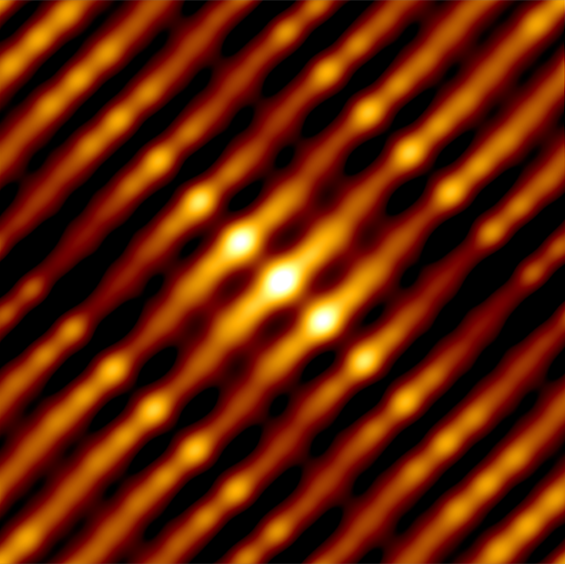

Supplement: Supplementary file 7 — Source Data [file 41467_2021_21838_MOESM7_ESM.zip › Source_Data/Figure2_Supplementary_Figure4a /Auto correlation/BUC topo 1 auto correlation.tif]

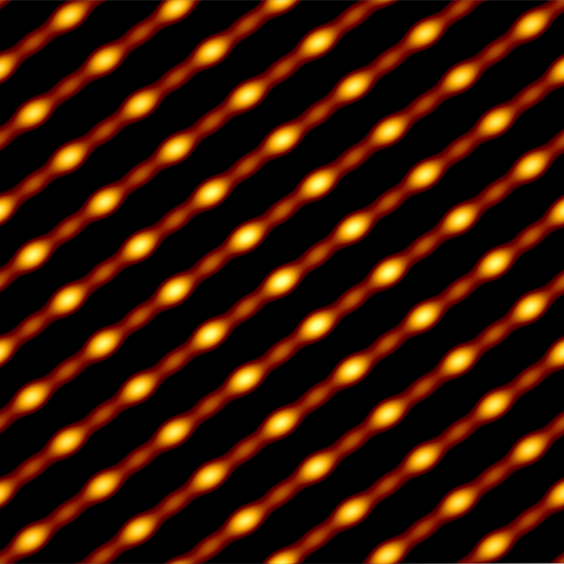

Supplement: Supplementary file 7 — Source Data [file 41467_2021_21838_MOESM7_ESM.zip › Source_Data/Figure2_Supplementary_Figure4a /Auto correlation/TUC 1 topo 1 auto correlation.tif]

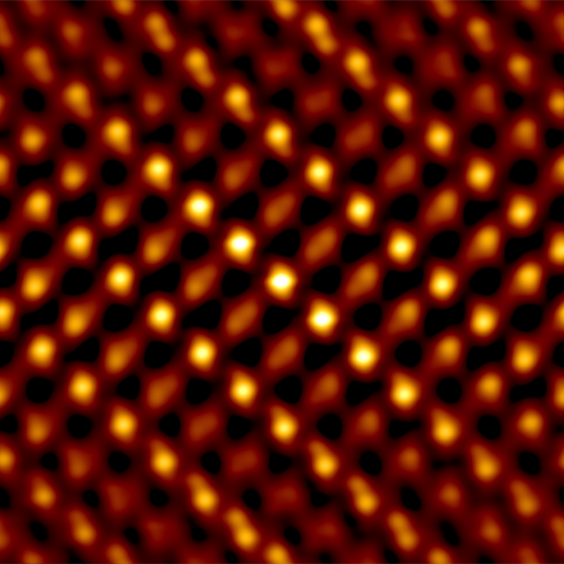

Supplement: Supplementary file 7 — Source Data [file 41467_2021_21838_MOESM7_ESM.zip › Source_Data/Figure2_Supplementary_Figure4a /Auto correlation/AUC topo 1 auto correlation .tif]

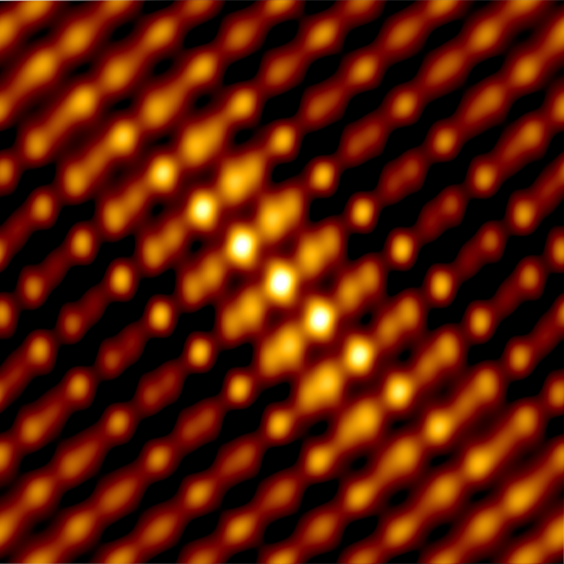

Supplement: Supplementary file 7 — Source Data [file 41467_2021_21838_MOESM7_ESM.zip › Source_Data/Figure2_Supplementary_Figure4a /Auto correlation/AUC topo 4 auto correlation.tif]

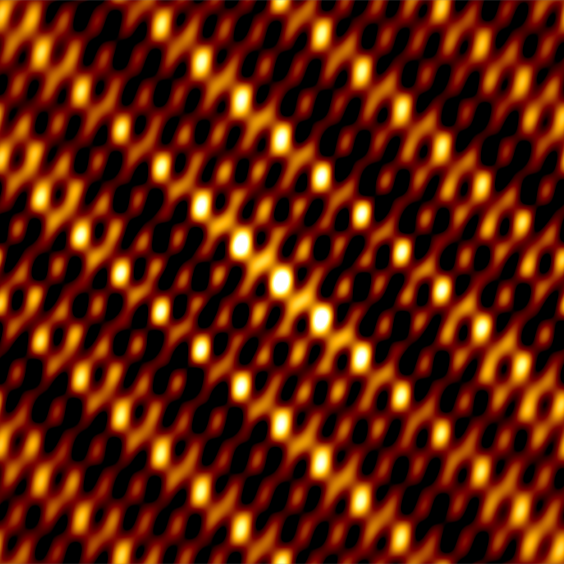

Supplement: Supplementary file 7 — Source Data [file 41467_2021_21838_MOESM7_ESM.zip › Source_Data/Figure2_Supplementary_Figure4a /Auto correlation/AUC topo 3 auto correlation.tif]

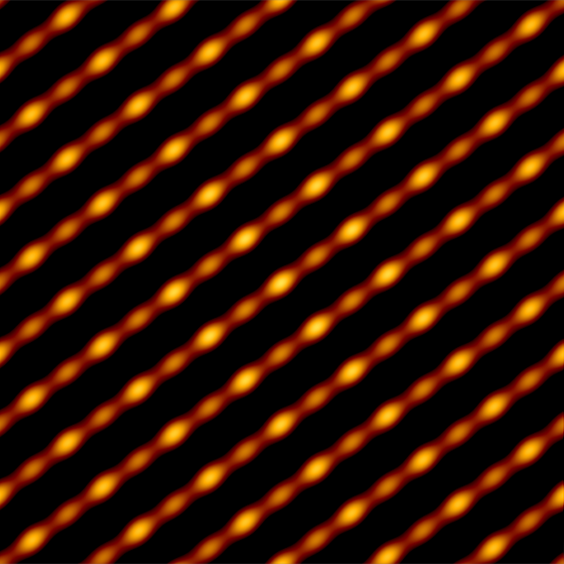

Supplement: Supplementary file 7 — Source Data [file 41467_2021_21838_MOESM7_ESM.zip › Source_Data/Figure2_Supplementary_Figure4a /Auto correlation/TUC 1 topo 2 auto correlation.tif]

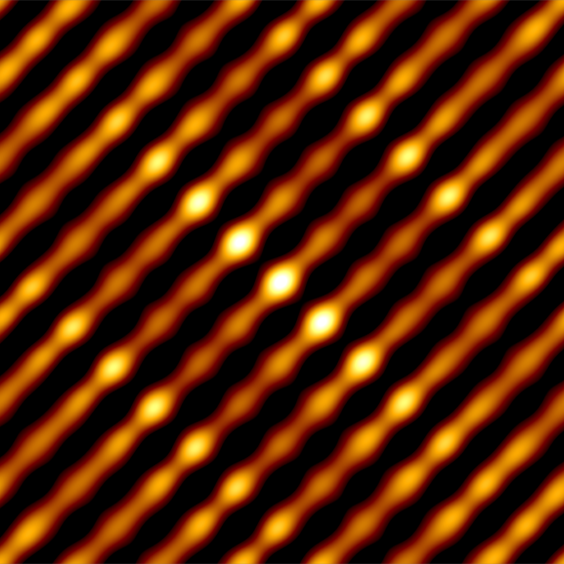

Supplement: Supplementary file 7 — Source Data [file 41467_2021_21838_MOESM7_ESM.zip › Source_Data/Figure2_Supplementary_Figure4a /Auto correlation/BUC topo 2 auto correlation.tif]

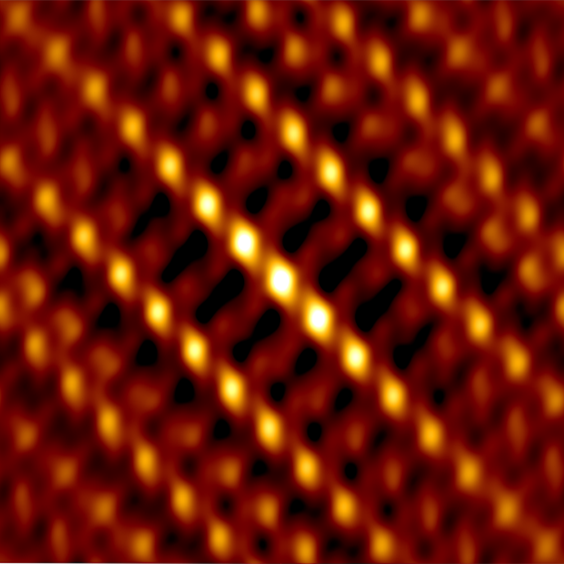

Supplement: Supplementary file 7 — Source Data [file 41467_2021_21838_MOESM7_ESM.zip › Source_Data/Figure2_Supplementary_Figure4a /Auto correlation/AUC topo 2 auto correlation.tif]

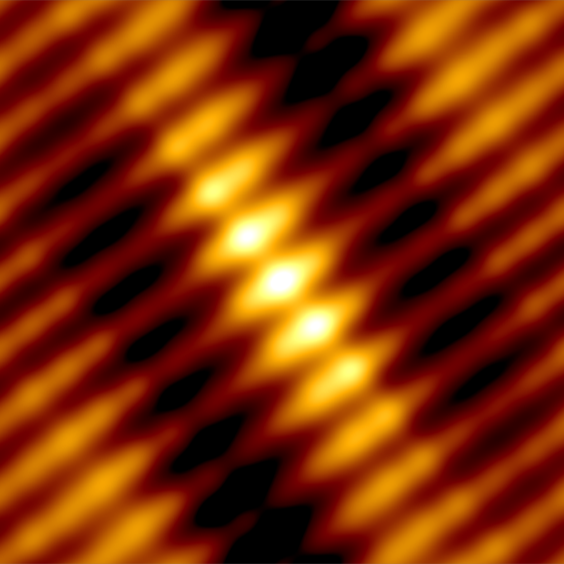

Supplement: Supplementary file 7 — Source Data [file 41467_2021_21838_MOESM7_ESM.zip › Source_Data/Figure2_Supplementary_Figure4a /Auto correlation/TUC 1 BUC intermediate auto correlation.tif]

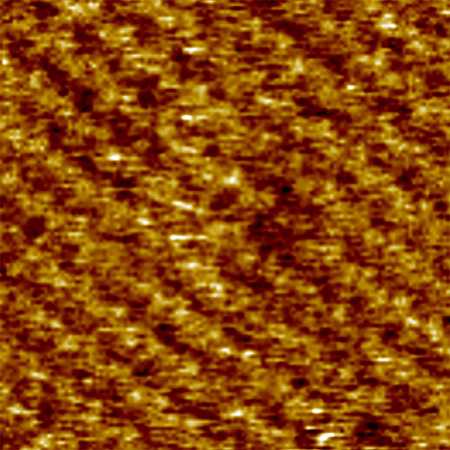

Supplement: Supplementary file 7 — Source Data [file 41467_2021_21838_MOESM7_ESM.zip › Source_Data/Supplementary_Figure4b/RAW AFM images /abAUC topo.tif]

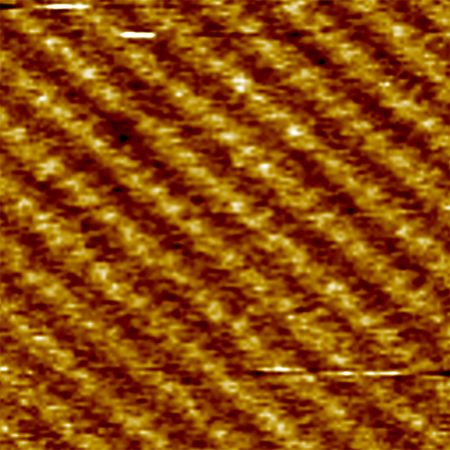

Supplement: Supplementary file 7 — Source Data [file 41467_2021_21838_MOESM7_ESM.zip › Source_Data/Supplementary_Figure4b/RAW AFM images /abBUC topo.tif]

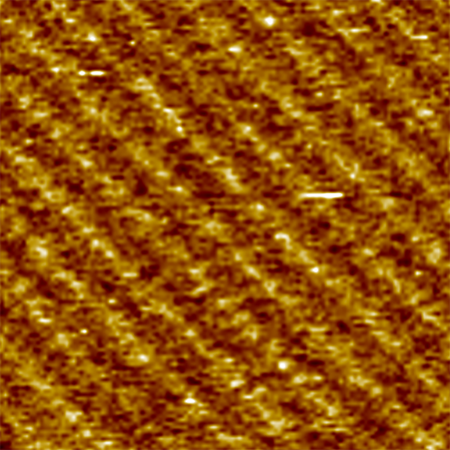

Supplement: Supplementary file 7 — Source Data [file 41467_2021_21838_MOESM7_ESM.zip › Source_Data/Supplementary_Figure4b/RAW AFM images /abTUC1 topo.tif]

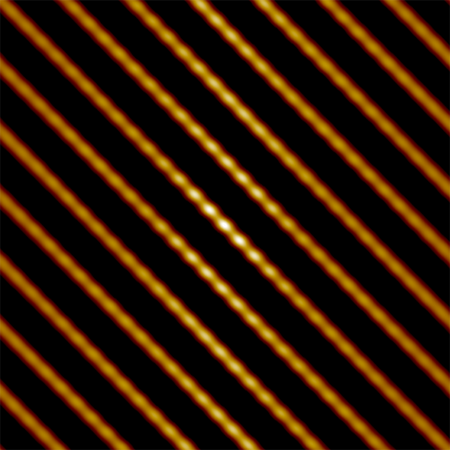

Supplement: Supplementary file 7 — Source Data [file 41467_2021_21838_MOESM7_ESM.zip › Source_Data/Supplementary_Figure4b/Auto correlation/abTUC1 topo auto correlation.tif]

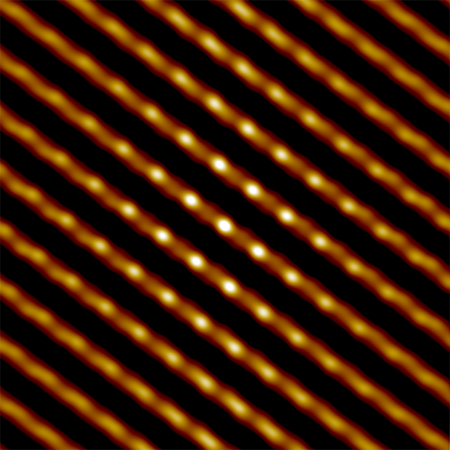

Supplement: Supplementary file 7 — Source Data [file 41467_2021_21838_MOESM7_ESM.zip › Source_Data/Supplementary_Figure4b/Auto correlation/abAUC topo auto correlation.tif]

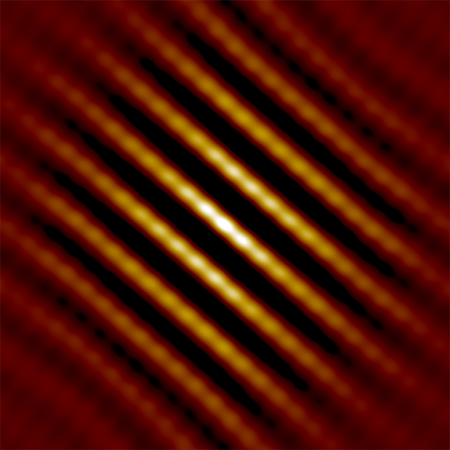

Supplement: Supplementary file 7 — Source Data [file 41467_2021_21838_MOESM7_ESM.zip › Source_Data/Supplementary_Figure4b/Auto correlation/abBUC topo auto correlation.tif]

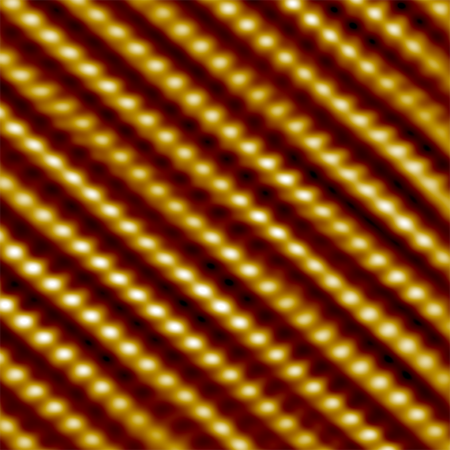

Supplement: Supplementary file 7 — Source Data [file 41467_2021_21838_MOESM7_ESM.zip › Source_Data/Supplementary_Figure4b/Filtered images/abBUC topo FIltered image.tif]

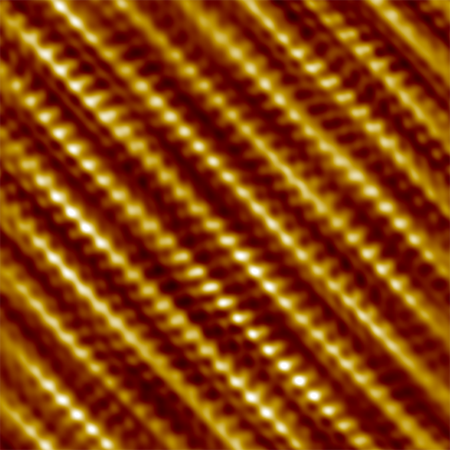

Supplement: Supplementary file 7 — Source Data [file 41467_2021_21838_MOESM7_ESM.zip › Source_Data/Supplementary_Figure4b/Filtered images/abTUC1 topo FIltered image.tif]

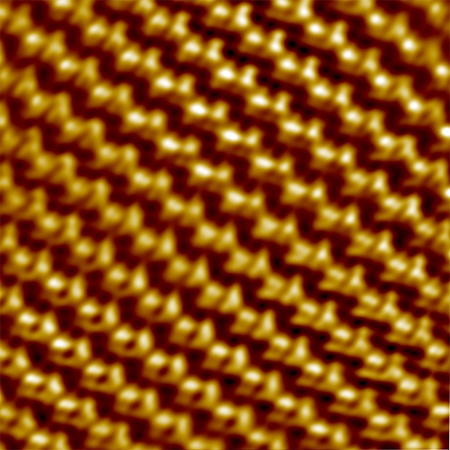

Supplement: Supplementary file 7 — Source Data [file 41467_2021_21838_MOESM7_ESM.zip › Source_Data/Supplementary_Figure4b/Filtered images/abAUC topo Filtered image.tif]

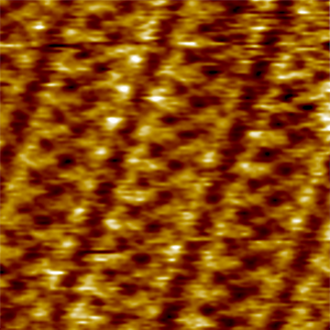

Supplement: Supplementary file 7 — Source Data [file 41467_2021_21838_MOESM7_ESM.zip › Source_Data/Supplementary_Figure4c/Raw AFM images/BUC topo 1 .tif]

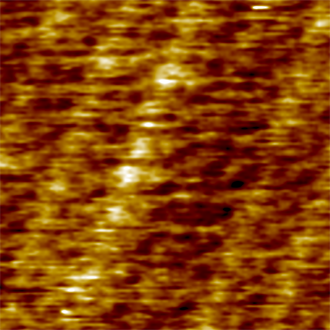

Supplement: Supplementary file 7 — Source Data [file 41467_2021_21838_MOESM7_ESM.zip › Source_Data/Supplementary_Figure4c/Raw AFM images/AUC topo 1 .tif]

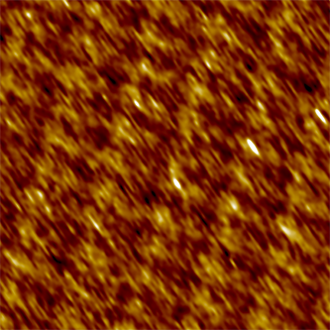

Supplement: Supplementary file 7 — Source Data [file 41467_2021_21838_MOESM7_ESM.zip › Source_Data/Supplementary_Figure4c/Raw AFM images/AUCrev topo 1.tif]

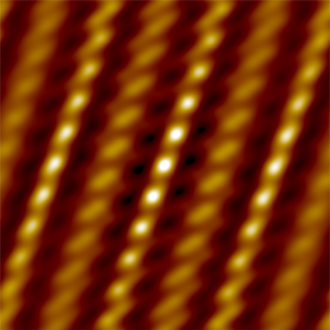

Supplement: Supplementary file 7 — Source Data [file 41467_2021_21838_MOESM7_ESM.zip › Source_Data/Supplementary_Figure4c/Auto correlation/BUC topo 1 auto correlation.tif]

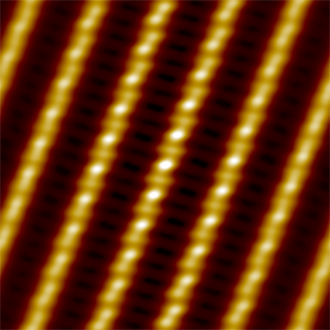

Supplement: Supplementary file 7 — Source Data [file 41467_2021_21838_MOESM7_ESM.zip › Source_Data/Supplementary_Figure4c/Auto correlation/AUC topo 1 auto correlation.tif]

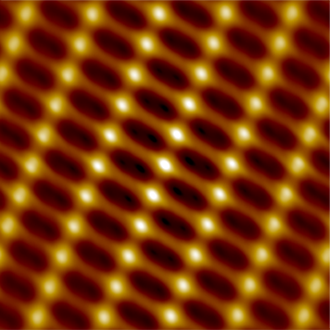

Supplement: Supplementary file 7 — Source Data [file 41467_2021_21838_MOESM7_ESM.zip › Source_Data/Supplementary_Figure4c/Auto correlation/AUCrev topo 1 auto correlation.tif]

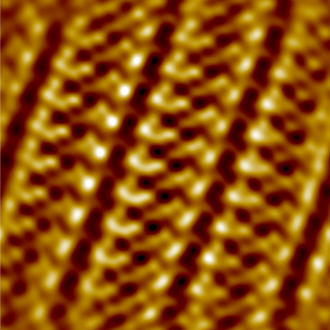

Supplement: Supplementary file 7 — Source Data [file 41467_2021_21838_MOESM7_ESM.zip › Source_Data/Supplementary_Figure4c/Filtered images/BUC topo 1 Filtered image.tif]

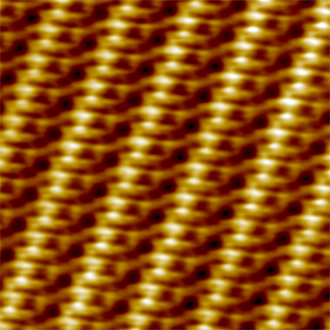

Supplement: Supplementary file 7 — Source Data [file 41467_2021_21838_MOESM7_ESM.zip › Source_Data/Supplementary_Figure4c/Filtered images/AUC topo 1 Filtered image .tif]

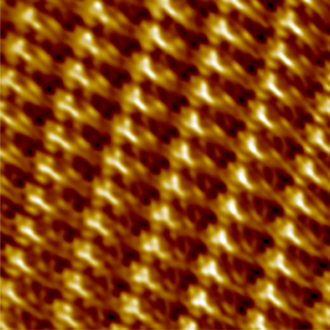

Supplement: Supplementary file 7 — Source Data [file 41467_2021_21838_MOESM7_ESM.zip › Source_Data/Supplementary_Figure4c/Filtered images/AUCrev topo 1 Filtered image.tif]
